# Investigating rehabilitation by activities involving the trunk to improve balance and gait control in young children with cerebral palsy: A randomized open-label crossover trial protocol

**DOI:** 10.1371/journal.pone.0334195

**Published:** 2026-03-13

**Authors:** Stella Zografou, Jonathan Pierret, Rajul Vasa, Jean Paysant, Christian Beyaert

**Affiliations:** 1 Centre de Médecine Physique et de Réadaptation de l’Enfance, Institut Régional de Réadaptation, UGECAM-NE, Flavigny-sur-Moselle, France; 2 Institut Régional de Réadaptation, UGECAM-NE, Nancy, France; 3 Université de Lorraine, DevAH research Unit, Development, Adaptation and Disability, Nancy, France; 4 RV Foundation, Centre for Brain and Spinal Injury Rehab, Mumbai, India; UFPE: Universidade Federal de Pernambuco, BRAZIL

## Abstract

**Introduction:**

Children with cerebral palsy (CP) have gross motor and balance disorders altering standing, walking and activities. Since trunk control is central for balance, rehabilitation targeting the trunk is developing. In children with CP aged 5–12 years, rehabilitation by activities involving the trunk (RAIT) based on activities in intermediate postures for 3 months has been demonstrated to significantly improve trunk control while standing and early trunk deceleration and coupled negative ankle power due to plantar flexors while walking autonomously. As motor disorders develop early, the effects of RAIT are investigated in younger children and for longer time: the adapted design of this study is presented. Initial motor disorders in children with CP aged 18 months to 5 years and 6 months are expected to show at least a 30% reduction after RAIT compared to UR (based on prior findings in older children), with progressive improvements of 40% and 50% at 6 and 12 months, respectively, reflecting cumulative training effects.

**Methods:**

The studied motor disorders include −1- during gait, excessive early anterior deceleration of the sternum (primary outcome) measured by inertial measurement unit, excessive anterior location of center of pressure on affected leg(s), Enhanced Gait Variability Index and step width measured by a walkway equipped with pressure sensors, −2- Altered gross motor, balance and trunk function measured by the item set version of the Gross Motor Function Measurement 66 and by the Early Clinical Assessment of Balance.

**Expected results:**

All these variables would be influenced by trunk balance and control, and therefore reduced after RAIT.

**Trial registration:**

ClinicalTrials.gov: NCT06438432.

## 1. Introduction

Cerebral Palsy (CP) is the most frequent disability of childhood that disturbs motor function with a prevalence of 2–3 births in every 1000 births [1]. Cerebral palsy is a group of permanent but not unchanging disorders of posture and movement which are attributed to non-progressive brain damage occurring during perinatal development [2,3]. The International Classification of Functioning, Disability and Health – children and youth version (ICF-CY) is a good way of addressing the impact of these deficits in children with CP. The ICF-CY provides a conceptual and systematic framework based on a biopsychosocial approach to standardize the health and health-related states of various pathological populations. Disability and function are described through the following three main domains: body functions and structures, activity and participation [4]. Cerebral palsy affects these three levels, with structural alterations of the neural and musculoskeletal systems affecting posture [5], activity and participation [6].

Indeed, postural control ensure safety balance against gravity and regulate the orientation and the position of body segments relative to the environment [7,8] in order to interact with the latter during voluntary movement. Its development relies on the proper maturation of the central nervous system [9], which is influenced by internal, such as the maturation of sensory information processing, and environmental constraints. These processes take place from the first year of life and continue during early childhood. Recent work demonstrates segmental trunk control during sitting strongly correlates with gross motor development in typically developing infants aged 4–12 months, with trunk control explaining 60–70% of variance in motor milestones [10].

The axial segments, in particular the trunk, play a key role in the development of postural control, with an impact on the different domains of the ICF. When trunk control is adequately developed, it provides a stable sitting posture [11] that allows for the development of gross motor function [10,11], manual skills [12,13] and interactions with others [14]. Later on, during childhood, spontaneous postural oscillations when standing decrease as the child grows. This decrease in oscillations is associated with improvement in postural control [15] where proactive control completes reactive control of disequilibrium [16] with an important part of trunk control [17].

Independent standing, which requires effective tonic antigravity activity of the trunk muscles, is an essential prerequisite for the onset of autonomous walking. The organization of the locomotor pattern, which emerges between 11 and 15 months [18,19], depends in large part on the progressive mastery of the coordination of axial segments during childhood [20–22]. The importance of trunk control during walking is present even in adulthood, contributing not only to the maintenance of locomotor balance but also to propulsion [23–27].

From infancy onwards, children with CP show impaired control of axial segments and particularly of the trunk. These disorders appear first in the sitting position with abnormal postural reaction compared to children with typical development (TD) [28] and persist throughout childhood with combined impairment of static and dynamic control, with probable disturbance of proactive control mechanisms [29–31]. The correlation between impaired trunk control and deficits in gross motor function has also been confirmed in children with CP [11].

Independent walking is a motor function that is crucial for autonomy, enabling the majority of activities of daily life to be carried out within the community. During walking, children with CP show deviations in head and trunk kinematics and kinetics in all three planes compared to children with TD [32–36]. These deviations are associated with altered balance control during walking (1), leading to greater step width and variability in step length [37,38] and increased accelerations of the axial segments in all three planes [39,40]. The latter is significantly related to trunk control deficit [41,42] that is the primary contributor to impaired walking performance in children with cerebral palsy, above neuromuscular deficits [43]. Since the trunk and lower limbs interact reciprocally during walking, deviations of the trunk can induce deviations of the lower limbs, and vice versa [41].

Toe walking, characterized by the absence of the first heel pivot from initial contact [44] is common in children with CP. During weight acceptance (WA) phase of gait (defined as the period of combined initial power absorption activity around the lower joints [45], toe walking is associated with an early ankle power absorption and negative work, resulting in early decelerated ankle dorsiflexion and anterior tibial tilt [46]. While the spastic origin of toe walking, i.e., related to hyperexcitable stretch reflexes, has been debated for decades and is now considered unlikely [47] authors have proposed this walking pattern to be part of an adaptive process [46–48]. Lorentzen and colleagues [49] demonstrated that during voluntary toe walking pattern the plantar flexors are activated before ground contact and remain engaged during the early stance phase, independently of peripheral sensory input. Given that the role of plantar flexors during WA is mainly to control trunk and body support and balance through their action on the tibia as soon as the foot is flat on the floor [50–52] toe walking allows the plantar flexors to early control the trunk from initial contact, suggesting their potential role to compensate for trunk control deficits that are associated to trunk lower stability [42]. Such a feedforward control mechanism allows the plantar flexors to anticipate the sensory consequences of each step and contribute to trunk stabilization, rather than being the result of hyperactive stretch reflexes [49]. This mechanism reinforces the view that toe walking can be an adaptive motor strategy, compensating for limitations in trunk stability or balance during gait. The prolonged early activation of plantar flexors during toe walking likely drives structural adaptations in the muscle-tendon complex, including increased connective tissue stiffness and altered sarcomere length, which passively contribute to early stance stability [53]. This structural remodeling represents a biomechanical adaptation to compensate for trunk control deficits rather than purely spastic pathology. Moreover, in a recent report from our research team [54], this adaptive role of the plantar flexors has been supported −1- by the significant correlation between negative ankle power and both the anterior deceleration of the upper trunk and the downward deceleration of the sacrum during WA; and −2- the simultaneous reduction in the upper trunk and sacrum deceleration and the negative ankle work during WA following trunk-focused rehabilitation resulting in significant improvement in trunk control.

Considering the significant role of the trunk in static and dynamic activities, as well as its impact on gross motor function from early childhood, rehabilitation targeting the trunk is being explored in children with CP [55–57] but its long-term outcomes remain unexplored. In children with CP aged 5–12 years walking autonomously, trunk-focused rehabilitation (RAIT) but not usual rehabilitation (UR), for 3 months each, significantly improved trunk control while sitting and standing and early trunk deceleration and coupled negative ankle power due to plantar flexors [54]. As these findings are promising, and since trunk control deficits appear early in children with CP, it is worth studying the effects of RAIT applied in younger children and for longer time. However, the protocol of this study needs specific adaptations in the content of RAIT and in technical aspects of assessing trunk control, gross motor control and gait dynamics. The aims and protocol of this project are presented and discussed below.

The aims of this randomized open-label crossover trial are to evaluate the motor effects of RAIT along its 9–12 months application and comparatively to usual rehabilitation (UR) during its first 3 months application in young children with CP who walk independently or with inconsistent use of a walking aid. Thus, initial motor disorders in children with CP aged 18 months to 5 years and 6 months, compared with children with TD, are expected to be better reduced after RAIT than after UR, and to be increasingly reduced after 3, 6 and 12 months of RAIT. Motor evaluation is carried out with consideration for the young age of the children. Trunk analysis during gait and gait analysis are based on inertial measurement units (IMUs) placed on the trunk for upper and lower trunk acceleration, on the use of a walkway equipped with pressure sensors for temporo-spatial parameters and center of pressure of the feet, and video recording for the Edinburgh visual gait score [58]. The Item Set version of the Gross Motor Function Measurement 66 (GMFM-66-IS) [59] and the Early Clinical Assessment of Balance (ECAB) [60] are used to assess gross motor function and early balance and trunk function, respectively.

Initial motor disorders in young children with CP compared to children with TD are hypothesized to affect: −1- balance and trunk control and gross motor function: decrease in ECAB and GMFM-66-IS scores; −2- trunk dynamics during WA of gait: increase in anterior deceleration of the sternum (primary outcome) and downward deceleration of the waist at the level of the L5 vertebra (closed to body center of mass); −3- gait pattern related to balance disorder: increase in the enhanced gait variability index (eGVI) and in step width; −4- gait pattern including a toe walking: excessive anterior location of center of pressure (CoP) during WA and decrease in the Edinburgh Visual Gait Score.

As balance disorders with a primary role of trunk control deficit are believed to be essential factors of these motor disorders in children with CP, we hypothesize a significant correlation between all these variables. For the same reason, all these variables are expected to better improve after RAIT than after UR, and to increasingly improve after 3, 6 and 12 months of RAIT.

In addition, as upper limb(s) function may be affected in children with CP with consequences in child’s activity and participation, the parents are asked to complete the Reach Out Questionnaire that allows a holistic overview of functioning including activity limitations and participation [61]. In our experience in children with hemiplegic and triplegic CP, RAIT improved the functional use of hand and upper limb and the child’s participation in various activities involving the upper limb. Thus, the Reach Out Questionnaire score is hypothesized to be reduced in children with CP compared to children with TD and to be increased more significantly after RAIT than after UR and further increased as RAIT is prolonged.

## 2. Materials and methods

### 2.1. Ethics

**Fig 1:**
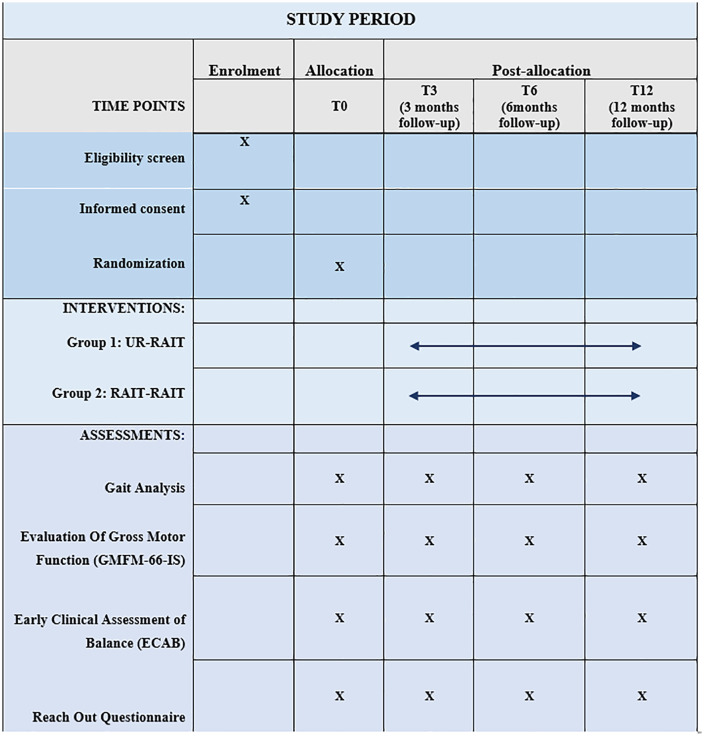
Time schedule of enrolment, interventions, and assessments. Overview of enrollment, intervention, and outcome assessments for participants, in accordance with SPIRIT 2013 guideline recommendations.

The experimental protocol complied with the tenets of the Declaration of Helsinki was approved by the French ethic committee (Comité de Protection des Personnes EST-I, numéro SI: 24.00651.000276) as required by French legislation. The ethics committee approved the study on 11 April 2024. This clinical trial is registered on ClinicalTrials.gov (reference: **NCT06438432**) and was developed in accordance with SPIRIT guidelines [62]. Further information on enrollment, interventions and assessments in (Fig 1).

The anonymity of participants will be ensured by assigning them a unique alphanumeric reference. This reference will be used to design the participant in any files containing the data to be analyzed. Only the principal investigator and his first collaborator (CB, SZ) have access to the folder containing the conversion key. In practice, the security of the anonymity of participants is not possible due to the close collaboration between the health care professionals and the patients. However, quantitative results will be presented in accumulated form, and care will be taken to guarantee that no participants are recognizable in the results. All data from assessments and rehabilitation participation will be securely stored and the patients deleted after completion of the study.

### 2.2. Participant enrolment and study timeline

The children are recruited and evaluated at the Centre de Médecine Physique et de Réadaptation de l’Enfance (CMPRE) in Flavigny-sur-Moselle, Lorraine, France. This is a regional center providing a range of functional assessment and treatment services for children with orthopedic, neurological or neuro-orthopedic abnormalities. The children with CP recruited may be part of cohorts of patients followed at the CMPRE or be referred by pediatricians, neuro-pediatricians and physical medicine and rehabilitation physicians from different networks interacting with the CMPRE. During an initial medical consultation with a physician working in collaboration with the CMPRE, if the child is eligible, their parents are informed about this study, receive an information and consent form, and are referred to the CMPRE for an assessment of their motor skills and a medical consultation. The physiotherapist treating their child will then be informed about the study and invited to collaborate. During the second medical consultation with the principal investigator (CB) at the CMPRE, the child will be offered the opportunity to participate in the study, if their eligibility is confirmed and if the physiotherapist and parents have given their written consent. If the child is enrolled, the therapeutic group assignment contained in a sealed envelope is revealed by the principal investigator (CB) and the motor skills assessments from that day are used as baseline assessments (M0).

For children with TD, recruitment will be conducted through an email announcement presenting the study and including the information and consent form. The email will be sent to staff at the Regional Rehabilitation Institute, the Nancy Regional and University Hospital Center, and the University of Lorraine, who reside in the Nancy metropolitan area and have no hierarchical relationship with the study investigators. As with the children with CP, they will be seen in consultation by an investigating physician.

The duration of patient participation is 12 months. Patient enrolment started on 25 April 2024 and will end on 31 October 2026. This enrolment period of 30 months is in line with the usual recruitment and follow-up of children with CP by the CMPRE. The total planned duration of the study (participation and data analysis) is 54 months.

### 2.3. Study design

This protocol is a monocentric open-label randomized cross over study with two groups of children with CP pre-per and post interventional rehabilitation. The control group of children with TD will be evaluated once and compared to children with PC at their initial period of evaluation (M0), carried out after recruitment. Children with CP are followed for 12 months, with evaluations at 3, 6 and 12 months after M0.

**Fig 2:**
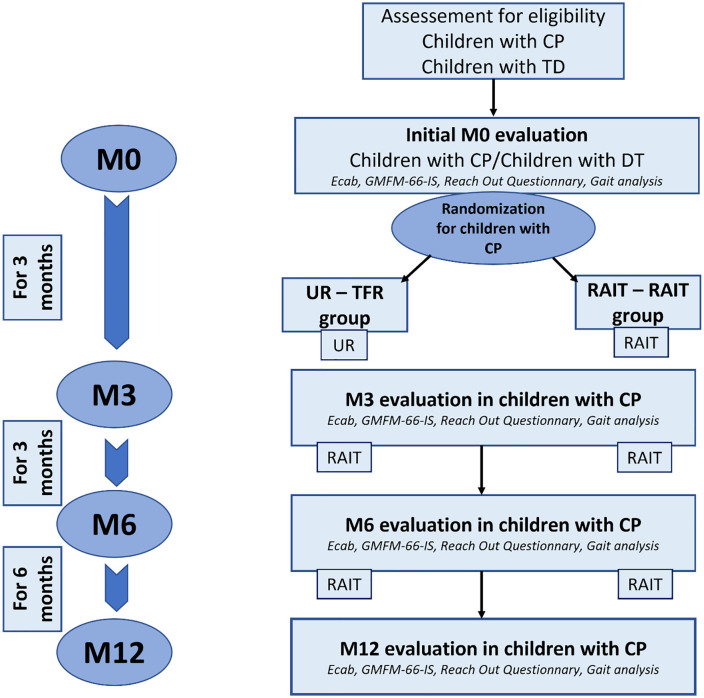
Study design. Children with cerebral palsy (CP) are randomly allocated to Usual Rehabilitation (UR) – Rehabilitation by Activities Involving the Trunk (RAIT) or RAIT – RAIT group. In the UR – RAIT group, UR is applied for 3 months and RAIT for the following 9 months. In the RAIT – RAIT group, RAIT is applied for 12 months. The children with CP are evaluated before the start of the rehabilitation program (at M0), after 3 months (M3), 6 months (M6) and 12 months (M12) of rehabilitation. A group of children with TD is evaluated once for comparison with the children with CP at M0. Ecab: Early Assessment Clinical Balance – GMFM-66-IS: Item Set version of the Gross Motor Function Measurement 66.

Children are randomly divided into two groups: In the first group (UR-RAIT), UR received by children is continued during the first 3 months, and is replaced by RAIT for the following 9 months. In the second group (RAIT-RAIT), children benefit from RAIT for the entire duration of the study. An individual random allocation of participants into the two groups in blocks of four using Matlab software was carried out in advance, then placed in sealed, numbered envelopes to be used in the order of inclusion. The study design is depicted in (Fig 2).

Given that UR is expected to have minimal effect on trunk control based on our prior findings, and that RAIT effects are hypothesized to be progressive and sustained, we assume minimal carryover from the UR period into the RAIT period in the UR-RAIT group. We will formally test the no-carryover assumption using Grizzle’s test (p < 0.10 threshold given low power). If carryover is detected, we will: (1) analyze only Period 1 as parallel groups (primary analysis), and (2) report full crossover results as secondary, acknowledging potential bias. Treatment-by-period interaction will be examined graphically and statistically.

This is a randomized controlled trial protocol, not a systematic review or meta-analysis. Therefore, PROSPERO registration is not applicable. The trial is registered on ClinicalTrials.gov (NCT06438432) in accordance with ICMJE requirements.

### 2.4. Participants

#### 2.4.1. Children with CP.

The main inclusion criteria for the children with CP are as follows: age between 18 months and 5 years and 6 months; ability to walk without aids for 7 meters (GMFCS level I or II) [63]; ankle dorsiflexion range of motion of at least 5° with knee extended (measured with goniometer, average of three trials); and presence of soleus spasticity defined as Modified Tardieu Scale R2-R1 ≥ 10° with knee flexed, assessed by trained physiotherapist with established inter-rater reliability (κ > 0.75) [64].

The main exclusion criteria are as follows: botulinum toxin injections in the lower limbs in the 6 months preceding the study; lower limb surgery in the 12 months preceding the study; any changes in physical or orthopedic therapy within the previous 2 months; minimum hip flexion greater than 20°; pain in the lower limbs when standing or walking; Inability to follow simple 1-step commands or severe behavioral disturbances preventing assessment completion, as judged by assessing clinician.

#### 2.4.2. Children with TD.

The children with TD will be age-matched with the children with TD. They must be able to walk independently at 18 months of age, to have sufficient cognitive level and cooperation to perform the evaluations, with no history of neurological or orthopedic disease, no history of lower limb surgery, and no pain.

### 2.5. Evaluations

#### 2.5.1. Gait analysis.

Gait analysis is threefold: analysis of trunk accelerations using IMUs, analysis of temporospatial gait parameters and centers of pressure using a walkway equipped with pressure sensors, and analysis of the Edinburgh visual gait score using video recording.

**2.5.1.1 Trunk dynamics:** Trunk accelerations are measured by Inertial Measurement Units (mTest3 system, mHealth Technologies Srl, Bologna, Italy), validated for trunk postural assessment during gait in adults and children [39]. Each IMU contains a tri-axial accelerometer (±16g range, 100 Hz sampling frequency, adequate for capturing peak accelerations during weight acceptance which occur at 5–8 Hz [26]. Units are attached to skin using hypoallergenic double-sided adhesive tape (3M™ Tegaderm™, 3M Health Care, St. Paul, MN, USA) at sternum, L5 level, and bilateral feet. Data are processed using a custom-made script on Matlab R2022b (MathWorks, Inc., Natick, MA, USA). The raw data are filtered using a 10 Hz low-pass filter and averaged over all gait cycles.

Peak anterior deceleration of the sternum during WA is the primary outcome of the study. High value of this variable is suggested to reflect the higher need to break the upper trunk forward progression during WA in order to compensate for insufficient postural control of the trunk [54]. Peak downward deceleration of L5 during WA is a secondary outcome: high value is suggested to reflect the higher need to break the downward movement of body center of mass during WA in order to compensate for insufficient balance and trunk control [21,54].

**2.5.1.2 Temporospatial parameters and trajectory of CoP:** Gait pattern related to balance disorder and to toe walking are obtained by a Zeno Walkway Gait Analysis System ®) equipped with pressure sensors positioned on its surface (427 cm x 122 cm, sampling frequency of 120 Hz). These sensors detect plantar pressures during walking, allowing to assess foot prints, trajectory of CoP and temporospatial parameters, processed by PKMAS software (ProtoKinetics Movement Analysis Software). The children are asked to walk barefoot back and forth along the 7 meters-long track several times, at spontaneous speed. Series of successive gait cycles during which the child is not distracted and walks in line with the treadmill are selected in order to obtain a total of more than 17 gait cycles for each side. Zeno Walkway (ProtoKinetics LLC, Havertown, PA, USA; 1.22 × 4.27 m active area, 18,432 sensors, 120 Hz sampling), validated for temporo-spatial analysis in children aged 2–18 years with excellent reliability (ICC > 0.90) [65].

The enhanced gait variability index (eGVI) is hypothesized to be increased. It is a composite score based on 9 temporospatial parameters that quantifies the distance between the amount of variability observed in an asymptomatic reference group and the amount of variability observed in the patient [66,67]. Indeed, this index that assesses instability during gait and the risk of falling is usually high in children with CP, as they are impacted by balance disorders [68,69].

Step width is hypothesized to increase. Indeed, this variable reflects a strategy for reducing the risk of falls in unstable gait and is usually high in children with CP [70].

Center of pressure location normalized to foot length during WA, on the affected side(s), is hypothesized to be excessively anterior (>0.70 of foot length from heel) in children with CP compared to children with TD (<0.60). This will be calculated as the mean anterior-posterior CoP position during the first 20% of stance phase, expressed as a proportion of total foot length. CoP calculation employs center-of-pressure algorithm in PKMAS software (v3.8.8), with foot segmentation based on pressure threshold of 5 N/cm² to distinguish loading phases.

**2.5.1.3 Visual evaluation of the gait kinematic pattern:** The Edinburgh Visual Gait Score, a kinematic gait pattern score based on two cameras recording front and side view, is hypothesized to be decreased as the gait pattern in CP children, including toe walking, is different from children with TD [58].

#### 2.5.2. Evaluation of gross motor function by clinical scores.

The Item Set of the Gross Motor Function Measurement 66 (GMFM-66-IS) is hypothesized to be reduced with CP. Compared to the standardized 66-items used to assess gross motor function [71] the item set version (GMFM-66-IS) which has the same validity is faster to realize (around 20–30 minutes versus 60–80 minutes) since it uses 15–39 items selected according to the achievement of 3 main items [72]. Thus, the GMFM-66-IS by saving time is of great interest for the young children of this study. An experienced assessor, who has initially completed the standardized GMFM-66-IS training (CanChild online modules), is responsible for conducting this assessment as part of the study.

The Early Clinical Assessment of Balance (ECAB) is hypothesized to be reduced in children with CP. This 13-item clinical scale assesses postural stability (balance ability) in children with cerebral palsy, with two subscales: one dedicated to head and trunk postural control, the other to sitting and standing postural control. This scale is validated with high reliability for children, for the different GMFCS levels [60,73,74]. The optimal score is 100.

#### 2.5.3. Evaluation of upper limb function, activity and participation.

Evaluation of upper limb function and child’s activity and participation is made by the Reach Out Questionnaire. This questionnaire filled by the parents ensure a holistic overview of functioning, when a hand and upper limb are affected, including the assessment of activity limitations, participation in activities, attitudes in different environments and satisfaction, according to the domains of the ICF [61]. Rehabilitation by activities involving the trunk is hypothesized to improve hand and upper limb function in case of disorders affecting the upper limb function and the child’s participation, since the trunk appears central in motor activities.

Therefore, by combining neuromuscular assessment, evaluation of overall body and trunk postural control, overall motor function, walking, as well as reaching, grasping behavior, and participation, our study evaluates the effect of RAIT on all ICF domains.

### 2.6. Intervention

If the children are randomly assigned to the first group (UR-RAIT), they will receive 3 months of UR followed by RAIT for the following 9 months. In the second group (RAIT-RAIT), children benefit from RAIT for the entire duration of the study.

#### 2.6.1. Rehabilitation by activities involving the trunk.

If the physiotherapist providing care for the child with CP agrees to participate in the study, he/she will be initially briefed on the principles of RAIT and given detailed instructions on its content shortly before the start of the RAIT treatment.

Rehabilitation by activities involving the trunk (RAIT) exploits the automatic postural control of body balance, an essential aspect of motor function that enables individuals to maintain balance during static postures or motor activities. It is also noteworthy that our rehabilitation protocol extends over a duration of up to one year, which appears to be an important factor for maintaining improvements in motor function. In our previous study in children with CP aged from 5 to 12 years, the RAIT was applied for 3 months with significant improvement of gait dynamics and pattern [54].

**Fig 3:**
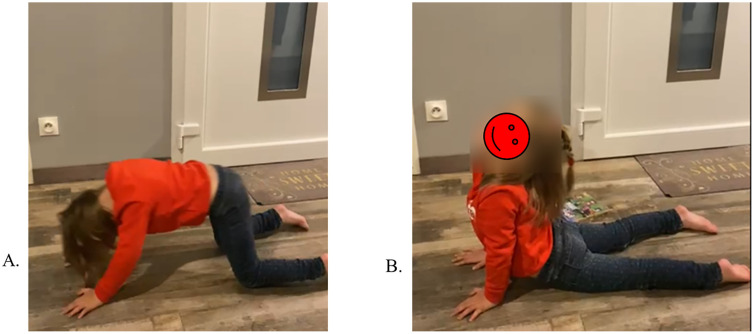
Examples of the proposed exercises involving the trunk. **A:** “On all fours”, the upper limbs display increased activation of the shoulder muscles, with recruitment of the antigravity extensors, particularly in the elbows and wrists, as well as a tendency for finger extension to allow the hands to lay flat on the ground. These effects are observed in both unaffected and affected upper limbs in children with cerebral palsy, as both contribute to postural balance. **B:** The transition to “Cobra” posture will promote higher support on the hands with strengthening of the elbow and wrist extensors. Elbow extension is facilitated by cervical extension (symmetrical tonic cervical extension reflex): the child is asked to look up when moving into the “Cobra” position. The Cobra posture is also accompanied by a noticeable extension of the hips.

The RAIT program focuses on self-directed exercises actively performed by the child, so that balance is controlled by the child and not by another person, in order to develop and engram balance strategies. These exercises aim to enhance body postural control and balance, including the trunk and affected muscles, rather than focusing rehabilitation on the affected muscles. In fact, RAIT does not only involve the trunk, but the entire body in selected intermediate postures involving the trunk that the child performs on his/her own (Fig 3). These postural activities are less challenging than standing or walking when considering balance control and help eliminate fear of falling. These activities allow automatic recruitment of deficient muscles through their contribution to balance control. For example, children with hemiplegic CP often have limited voluntary control of their affected upper limb: using the latter as support will automatically activate wrist and elbow extension and shoulder control without voluntary control of the affected muscles. Exercise progression follows standardized criteria: (1) child performs posture independently ×3 consecutive sessions, (2) maintains posture >30 seconds, (3) demonstrates correct form per checklist. Physiotherapists receive 2-hour training workshop with video examples and competency assessment before study initiation. Then, one of the first RAIT sessions in the physiotherapist’s office is conducted via videoconference in order to agree on its content.

**Fig 4:**
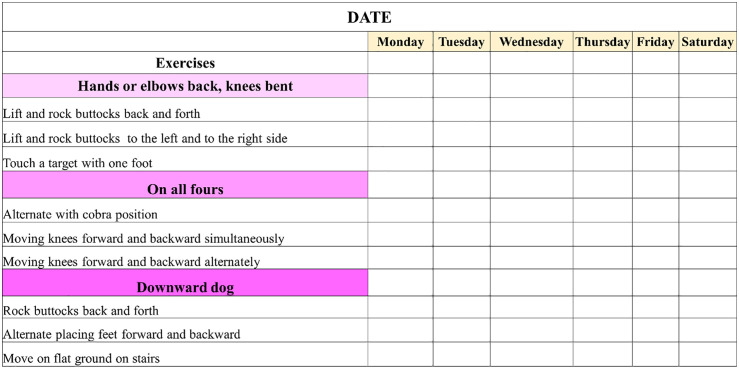
Part of a monitoring log in which a series of exercises are suggested. Part of a monitoring log in which a series of exercises are suggested: the physical therapist selects and adjusts some of them according to the child’s abilities, and the parents indicate which ones have been completed by the child.

Self-exercises are performed by the child, under the guidance of the physiotherapist, in separate small sessions, if possible, for a total of 20–30 minutes a day, 6 days a week, at home under the supervision of a parent and by the physiotherapist in one to two sessions per week. The use of a monitoring log, in which the physiotherapist notes down the exercises to be performed and the parents indicate those that have been completed, will allow for smoother and more regular monitoring of difficulties and progress in rehabilitation (Fig 4). Exercise compliance calculated as: (completed sessions/ prescribed sessions) × 100%. Target compliance ≥75% (≥18/24 weeks). Children <60% compliance will be analyzed separately as per-protocol sensitivity analysis.

#### 2.6.2. Usual rehabilitation.

The UR corresponds to the type of rehabilitation already received by the child before the study. It variously combines muscle stretching, muscle strengthening (e.g., resistance training), muscle tone reduction (e.g., Bobath concept neuro – developmental treatment), and upper and lower limb motor skill training facilitated by the therapist. These therapies usually involve limited groups of muscles in elementary stretching or actions. In the UR-RAIT group, the physiotherapist is asked to give to the parents an adapted selection of the usual rehabilitation to be achieved at home for a total duration of 20–30 minutes per day, the days without physiotherapist session, for a total of 6 days a week. A monitoring log, as previously described for RAIT, will be used with the exercises prescribed by the physiotherapist.

### 2.7. Data management

The study is conducted in accordance with the provisions of Law No. 78−17 of January 6, 1978, on information technology, files, and civil liberties, as amended by Law No. 2018−493 of June 20, 2018, on the protection of personal data, and European Regulation No. 2016/679 (GDPR). The reference methodology for the processing of personal data in the context of biomedical research applied in this study is MR-001.

Medical data concerning the children, and data necessary for the study will be collected and transmitted under the responsibility of the Research Promotor and its Data Protection Officer. The data collected during this study may be used for publication in scientific journals, and may be reused for other research in the field of trunk control and gait disorders in CP.

#### 2.7.1. Sample size.

Sample size was calculated for the primary outcome (peak anterior sternum deceleration during WA) using crossover design framework with Bonferroni adjustment for key secondary outcomes (n = 4 secondary: L5 deceleration, eGVI, step width, CoP location; adjusted α = 0.0125). Between-period correlation: Based on motor development literature in toddlers [75], we conservatively estimate ρ = 0.50 (lower than adult studies due to developmental variability). Variability estimate: Pilot data in 16 children with CP aged 1.5–5.5 years (unpublished) showed sternum deceleration SD = 1.10 m·s ⁻ ². We conservatively use SD = 1.20 m·s ⁻ ². These results are consistent with those of a study reporting maximum vertical acceleration of the pelvis at initial contact in children during the first 5 years of walking, where the standard deviation was < 0.8 m·s ⁻ ² at all times except around 6 months of walking, when it reached 1.5 m·s ⁻ ² [21].

Effect size: Based on prior RAIT study showing 1.20 m·s ⁻ ² reduction in older children, we expect minimum clinically important difference = 1.00 m·s ⁻ ² (30% reduction from baseline mean ≈ 3.30 m·s ⁻ ²) in younger population. Calculation: Two-sided paired t-test (α = 0.0125, power = 0.80, ρ = 0.50, MCID = 1.00, SD = 1.20) requires n = 17 per group. Accounting for 30% attrition (higher than typical due to: young age, 12-month duration, family relocation, illness), we plan n = 25 per group (50 total CP children).

Sample size calculated using G*Power 3.1.9.7, verified with PASS 2024 crossover module. For CP vs TD baseline comparison (independent t-test, d = 1.80, α = 0.05, power = 0.80): n = 12 per group required; we recruit n = 17 TD children for age-stratified matching (1:3 ratio).

#### 2.7.2. Data processing and quality control.

For gait analysis, raw IMU data (.csv) will be extracted from the mTest3 software and processed via a custom MATLAB script (version R2022b, available at [repository link upon publication]). Data relating to the walkway will be exported from PKMAS in.xlsx format and imported into R. The gait cycle will be identified based on the IMU signal from the foot (maximum vertical acceleration > 2 m·s ⁻ ²) = foot contact). The quality criteria for walking are as follows: ≥ 17 cycles per limb, continuous walking (no stopping/starting), and child looking forward (head rotation ± 30°).

### 2.8. Statistical analysis

#### 2.8.1. Primary analysis (intention-to-treat).

For primary outcome (sternum deceleration), we fit linear mixed-effects model: Y_ijk = μ + α_i + β_j + γ_k + δ_(jk) + π_t + (π_t × β_j) + τ_(period) + λ_(carryover) + ε_ijk. where **Y_ijk** is the outcome for child *i*, in sequence *j*, at assessment *k*; **α_i** is a random child effect ~ N(0, σ²_child), accounting for between-subject variability; **β_j** is the fixed sequence effect (UR–RAIT vs. RAIT–RAIT); **γ_k** is the fixed treatment effect (UR vs. RAIT), representing the primary parameter of interest; **δ_jk** is the sequence-by-treatment interaction; **π_t** is the fixed time effect across the four assessment points (M0, M3, M6, M12); **π_t × β_j** is the time-by-sequence interaction, capturing potential dose–response patterns; **τ_period** is the fixed period effect (first 3-month interval vs. later intervals); **λ_carryover** is the carryover effect, testing the validity of the crossover assumption; and **ε_ijk** is the residual error ~ N(0, σ²_error).

#### 2.8.2. Model assumptions.

Residual normality will be assessed using Q–Q plots and Shapiro–Wilk tests on residuals; homoscedasticity will be evaluated through residuals-versus-fitted plots and Levene’s test by group; and linearity will be examined using partial residual plots for continuous predictors. If assumptions are violated, log or Box–Cox transformations will be applied, or robust mixed models will be fitted using the rlmer package.

#### 2.8.3. Missing data.

Missing-at-random assumption will be tested using multiple imputation by chained equations (MICE package, m = 50 imputations, following Morris et al. 2014 guidelines for adequate precision). The imputation model will include baseline outcome, treatment allocation, age, sex, GMFCS level, CP subtype, ECAB score, and the number of attended sessions. A sensitivity analysis using complete-case analysis will be conducted to assess the plausibility of the missing-at-random assumption.

#### 2.8.4. Primary hypothesis test.

The primary comparison will test H₀: γ_RAIT − γ_UR = 0 versus H₁: γ_RAIT − γ_UR ≠ 0, using a Wald test on the γ contrast with a Bonferroni-adjusted significance level of α = 0.0125 (two-tailed).

#### 2.8.5 Carryover assessment.

The presence of carryover effects will be evaluated both graphically, using spaghetti plots by sequence showing individual trajectories, and statistically, by testing λ_carryover ≠ 0 using a likelihood ratio test. If p_carryover < 0.10, the analysis will revert to a Period 1 parallel-group analysis as the primary analysis, and the full crossover results will be reported as a sensitivity analysis.

#### 2.8.6. Secondary analyses.

**Dose–response.** The time-by-sequence interaction will be tested to determine whether the RAIT–RAIT group shows greater M0-to-M12 improvement than the UR–RAIT group, using linear contrasts with sequential Bonferroni correction.

**Reproducibility.** To assess the reproducibility of the RAIT effect, the first RAIT exposure in both groups will be compared (M0 → M3 in the RAIT–RAIT group vs. M3 → M6 in the UR–RAIT group) using a linear mixed model with a treatment-by-cohort interaction.

**Sustained effects.** Whether M12 outcomes differ from M6 within the RAIT–RAIT group will be tested using paired contrasts.

**Secondary outcomes.** The same linear mixed-effects model approach will be applied to L5 deceleration, eGVI, step width, centre of pressure location, GMFM-66-IS, ECAB, and Reach-Out scores, with Bonferroni adjustment (α = 0.0125 for the four gait outcomes).

#### 2.8.7. CP vs. TD comparison (M0).

Between-group comparisons at M0 will use independent t-tests (or Welch’s t-test if Levene’s test indicates unequal variances at p < 0.05). If normality is violated (Shapiro–Wilk p < 0.05), Mann–Whitney U tests will be used. Results will be reported as mean differences with 95% confidence intervals, Cohen’s d (or rank-biserial correlation for U-tests), and exact p-values.

#### 2.8.8. Correlation analyses (exploratory).

Within the CP group at M0, relationships between trunk dynamics, gait instability measures, and clinical scores will be examined using Pearson’s correlation coefficient (or Spearman’s rank correlation if normality assumptions are not met). Results will be reported as r with 95% confidence intervals and p-values, with Bonferroni correction for family-wise error (n = 10 correlations, adjusted α = 0.005).

#### 2.8.9. Software and reporting.

All analyses will be conducted using R version 4.3.0 or later with the following packages: lme4 (linear mixed models), lmerTest (p-values for mixed models), mice (multiple imputation), emmeans (post-hoc contrasts), and effectsize (Cohen’s d with confidence intervals). All estimates will be reported with 95% confidence intervals and exact p-values (e.g., p = 0.03, not p < 0.05). Effect sizes (Cohen’s d or partial η²) will be reported with 95% confidence intervals for all primary and secondary outcomes. Tables will include point estimate, standard error, 95% confidence interval, t/F-statistic, degrees of freedom, and p-value.

## 3. Discussion

Due to the high frequency of balance and postural disorders in children with CP [76] and in particular of trunk disorders [30,42] which affect the different domains of the ICF-CY [6,77], it is important to offer adequate rehabilitation programs targeting these specific deficits. Recent evidence has shown that segmental trunk control plays a crucial role in the acquisition of gross motor functions in children with CP [10], highlighting the growing importance of developing trunk stability from an early age [10]. Among trunk targeted interventions that develop for more than a decade [78–80] to our knowledge, this study self-distinguishes by the original approach and content of RAIT, by the young age of the children with CP and by the long-term application and evaluation of RAIT.

The RAIT considers balance control as a priority motor function that significantly impacts motor patterns and activities in upper motor neuron syndromes such as in cerebral palsy [81]. In these pathologies, the brainstem and cerebellum that support balance and postural control are usually anatomically intact [81]. In RAIT, the children perform functional exercises, i.e., self-directed activities based on intermediate postures involving the trunk and the four limbs and exploiting the automatic control of balance. In other words, the affected trunk and limbs will automatically participate to balance control according to their involvement to balance in intermediate postures. The intermediate postures are chosen to be securely performed with no fear of falling and to further involve the affected muscles in body balance. For example, being on all fours or in cobra posture would automatically recruit extensors of the different joints of the upper limbs to contribute to body balance **(see** Fig 2**)**. Activities in progressively more challenging intermediate postures for balance will be chosen to further increase the contribution of the trunk and affected limbs to balance in various motor activities. Therefore, an improvement in activities and participation according to the ICF-CY is expected after RAIT. The self-exercises of RAIT, carried out daily, are subject to individualized adjustments in line with the child’s abilities.

In addition, this study could provide new elements towards a rethinking of the pathophysiology of motor disorders during walking in children with CP. For example, recent studies question spasticity as the main explanatory factor for toe walking in children with CP [47,82]. One possible explanation is that it is an adaptive strategy [46,48] to compensate for a deficit in trunk control during walking. Toe walking is a feedforward process [48,49] allowing the plantar flexors to early control the trunk from initial contact [50–52]. Interestingly, adaptation to walking in negative heel shoes has been shown to be similar in typically developing children and children with cerebral palsy: it featured ankle dorsiflexion upon initial contact, even though (in the latter group) the soleus was always spastic in a clinical examination [46]. Hence, in children with cerebral palsy, the early deceleration of ankle dorsiflexion by the plantar flexors (promoted by early flattening of the foot, and regardless of the type of footwear) may have a functional role [46]. In addition, the early deceleration of ankle dorsiflexion would be reinforced passively by the development of connective tissue in the plantar flexors musculotendinous units in response to long term toe walking [53,83].

Thus, according to this pathophysiology, improvement in trunk control through RAIT is expected to lead to a reduction in compensation exerted by the foot: a reduction in the anterior deceleration of the sternum is expected to be associated to a reduction in the anterior shift of the CoP, during the WA of walking.

### 3.1. Limitations

This study presents several limitations mainly related to the young population of children with CP.

#### 3.1.1. Participation.

In longitudinal studies, involving young children can be particularly challenging, as fluctuations in the child’s level of cooperation, alertness or health may prevent regular involvement or completion of the rehabilitation program 6 days per week. Additionally, participation rates may be reduced by time constraints due to parents’ or school schedules.

#### 3.1.2. Developmental variability.

At young age (18 months to 5 and a half years of age), children’s development is highly variable, either for motor, cognitive, emotional, or social development. This natural variability linked to the young age of the children, combined with the variability in the severity of CP and the performance of RAIT, makes it difficult to estimate the number of subjects required for statistical purposes.

#### 3.1.3. Ethical considerations.

When conducting research in children, informed consent must be obtained from the parents or legal guardians, and if possible, from the child. In practice, due to the young age of children, communication with the child is mainly dedicated to the good realization of the rehabilitation content.

### 3.2. Perspectives

In case of good results, RAIT, which requires regular monitoring but little equipment and resources, may be to consider for routine clinical practice in children with CP. The self-exercises proposed, guided by the physiotherapist, would be carried out at home under the supervision of parents. Indeed, the active involvement of parents in their child’s care process appears to be a considerable asset for the success of this intensive approach for motor improvement including gait quality. Given the central role of the trunk in the development of body balance control, applying RAIT in young children with CP before they experience standing and walking would constitute the next step to be studied to reduce or avoid the development of compensatory mechanism by the lower limbs during standing and walking acquisition.

## Supporting information

S1 File RAIT in CP Protocol-FRENCH. (DOCX)

S2 File RAIT in CP Protocol-Translation ENGLISH. (DOCX)

**What this paper adds?**

- Balance control is essential in motor control with a central role of the trunk.
- Children with cerebral palsy (CP) have motor, balance and trunk deficits.
- A trunk-focused rehabilitation by activities in intermediate postures presented (RAIT).
- Study design to assess the long-term effects of RAIT in young children with CP.
- RAIT would improve trunk and foot dynamics during gait and gross motor function.

## References

[1] Oskoui M, Coutinho F, Dykeman J, Jetté N, Pringsheim T. An update on the prevalence of cerebral palsy: a systematic review and meta-analysis. Dev Med Child Neurol. 2013;55(6):509–19. doi: 10.1111/dmcn.12080 23346889

[2] Rosenbaum P, Paneth N, Leviton A, Goldstein M, Bax M, Damiano D, et al. A report: the definition and classification of cerebral palsy. Dev Med Child Neurol. 2006;49:8–14. doi: 10.1111/j.1469-8749.2007.tb12610.x 17370477

[3] Bax M, Goldstein M, Rosenbaum P, Leviton A, Paneth N, Dan B, et al. Proposed definition and classification of cerebral palsy. Dev Med Child Neurol. 2005;47:571–6. doi: 10.1017/S001216220500112X 16108461

[4] Organization WH. International Classification of Functioning Disability and Health (ICF). Geneva: World Health Organization; 2001.

[5] Woollacott MH, Burtner P. Neural and musculoskeletal contributions to the development of stance balance control in typical children and in children with cerebral palsy. Acta Paediatr Suppl. 1996;416:58–62. doi: 10.1111/j.1651-2227.1996.tb14279.x 8997450

[6] Kara OK, Gursen C, Cetin SY, Tascioglu EN, Muftuoglu S, Damiano DL. The effects of power exercises on body structure and function, activity and participation in children with cerebral palsy: an ICF-based systematic review. Disabil Rehabil. 2023;45(22):3705–18. doi: 10.1080/09638288.2022.2138575 36314560

[7] Massion J. Postural control system. Curr Opin Neurobiol. 1994;4(6):877–87. doi: 10.1016/0959-4388(94)90137-6 7888772

[8] Massion J, Alexandrov A, Frolov A. Why and how are posture and movement coordinated? Progress in Brain Research. Elsevier; 2004. pp. 13–27. doi: 10.1016/S0079-6123(03)43002-1 14653147

[9] Assaiante C, Mallau S, Viel S, Jover M, Schmitz C. Development of postural control in healthy children: a functional approach. Neural Plast. 2005;12(2–3):109–18; discussion 263-72. doi: 10.1155/NP.2005.109 16097479 PMC2565455

[10] Curtis DJ, Butler P, Saavedra S, Bencke J, Kallemose T, Sonne-Holm S, et al. The central role of trunk control in the gross motor function of children with cerebral palsy: a retrospective cross-sectional study. Dev Med Child Neurol. 2015;57(4):351–7. doi: 10.1111/dmcn.12641 25412902

[11] Pin TW, Butler PB, Cheung H-M, Shum SL-F. Relationship between segmental trunk control and gross motor development in typically developing infants aged from 4 to 12 months: a pilot study. BMC Pediatr. 2019;19(1):425. doi: 10.1186/s12887-019-1791-1 31711441 PMC6844031

[12] Butler PB. A preliminary report on the effectiveness of trunk targeting in achieving independent sitting balance in children with cerebral palsy. Clin Rehabil. 1998;12(4):281–93. doi: 10.1191/026921598667577442 9744664

[13] de Graaf-Peters VB, Blauw-Hospers CH, Dirks T, Bakker H, Bos AF, Hadders-Algra M. Development of postural control in typically developing children and children with cerebral palsy: possibilities for intervention? Neurosci Biobehav Rev. 2007;31(8):1191–200. doi: 10.1016/j.neubiorev.2007.04.008 17568673

[14] Kyvelidou A, Harbourne RT, Willett SL, Stergiou N. Sitting postural control in infants with typical development, motor delay, or cerebral palsy. Pediatr Phys Ther. 2013;25(1):46–51. doi: 10.1097/PEP.0b013e318277f157 23288009

[15] Cignetti F, Chabeauti P-Y, Sveistrup H, Vaugoyeau M, Assaiante C. Updating process of internal models of action as assessed from motor and postural strategies in children. Neuroscience. 2013;233:127–38. doi: 10.1016/j.neuroscience.2012.12.040 23291457

[16] Rival C, Ceyte H, Olivier I. Developmental changes of static standing balance in children. Neurosci Lett. 2005;376(2):133–6. doi: 10.1016/j.neulet.2004.11.042 15698935

[17] Pierret J, Beyaert C, Paysant J, Caudron S. How do children aged 6 to 11 stabilize themselves on an unstable sitting device? The progressive development of axial segment control. Hum Mov Sci. 2020;71:102624. doi: 10.1016/j.humov.2020.102624 32452427

[18] Malina RM. Motor Development during infancy and early childhood: overview and suggested directions for research. Int J Sport Health Sci. 2004;2:50–66. doi: 10.5432/ijshs.2.50

[19] Adolph KE, Vereijken B, Shrout PE. What changes in infant walking and why. Child Dev. 2003;74(2):475–97. doi: 10.1111/1467-8624.7402011 12705568

[20] Bril B, Dupuy L, Dietrich G, Corbetta D. Learning to tune the antero-posterior propulsive forces during walking: a necessary skill for mastering upright locomotion in toddlers. Exp Brain Res. 2015;233(10):2903–12. doi: 10.1007/s00221-015-4378-6 26246420

[21] Brenière Y, Bril B. Development of postural control of gravity forces in children during the first 5 years of walking. Exp Brain Res. 1998;121(3):255–62. doi: 10.1007/s002210050458 9746131

[22] Assaiante C. Development of locomotor balance control in healthy children. Neurosci Biobehav Rev. 1998;22(4):527–32. doi: 10.1016/s0149-7634(97)00040-7 9595565

[23] Cappozzo A. The forces and couples in the human trunk during level walking. J Biomech. 1983;16(4):265–77. doi: 10.1016/0021-9290(83)90134-3 6863342

[24] Cromwell RL, Aadland-Monahan TK, Nelson AT, Stern-Sylvestre SM, Seder B. Sagittal plane analysis of head, neck, and trunk kinematics and electromyographic activity during locomotion. J Orthop Sports Phys Ther. 2001;31(5):255–62. doi: 10.2519/jospt.2001.31.5.255 11352192

[25] Honeine J-L, Schieppati M, Gagey O, Do M-C. By counteracting gravity, triceps surae sets both kinematics and kinetics of gait. Physiol Rep. 2014;2(2):e00229. doi: 10.1002/phy2.229 24744898 PMC3966244

[26] Kavanagh J, Barrett R, Morrison S. The role of the neck and trunk in facilitating head stability during walking. Exp Brain Res. 2006;172(4):454–63. doi: 10.1007/s00221-006-0353-6 16489437

[27] Winter D. Human balance and posture control during standing and walking. Gait Posture. 1995;3(4):193–214. doi: 10.1016/0966-6362(96)82849-9

[28] Brogren E, Hadders-Algra M, Forssberg H. Postural control in sitting children with cerebral palsy. Neurosci Biobehav Rev. 1998;22(4):591–6. doi: 10.1016/s0149-7634(97)00049-3 9595574

[29] Heyrman L, Desloovere K, Molenaers G, Verheyden G, Klingels K, Monbaliu E, et al. Clinical characteristics of impaired trunk control in children with spastic cerebral palsy. Res Dev Disabil. 2013;34(1):327–34. doi: 10.1016/j.ridd.2012.08.015 23000634

[30] Pierret J, Caudron S, Paysant J, Beyaert C. Impaired postural control of axial segments in children with cerebral palsy. Gait Posture. 2021;86:266–72. doi: 10.1016/j.gaitpost.2021.03.012 33819768

[31] Saavedra SL, Woollacott MH. Segmental contributions to trunk control in children with moderate-to-severe cerebral palsy. Arch Phys Med Rehabil. 2015;96(6):1088–97. doi: 10.1016/j.apmr.2015.01.016 25656342 PMC4457569

[32] Attias M, Bonnefoy-Mazure A, Lempereur M, Lascombes P, De Coulon G, Armand S. Trunk movements during gait in cerebral palsy. Clin Biomech (Bristol). 2015;30(1):28–32. doi: 10.1016/j.clinbiomech.2014.11.009 25480360

[33] Hazari A, Agouris I, Wakode PS, Jadhav RA, Sharma N, Jena S, et al. Head and trunk kinematics and kinetics in normal and cerebral palsy gait: a systematic review. Eur J Physiother. 2020;22:168–77. doi: 10.1080/21679169.2019.1573919

[34] Kiernan D. The relationship of trunk kinematics and kinetics with lower limb pathology during gait in children with spastic cerebral palsy. Gait Posture. 2021;86:33–7. doi: 10.1016/j.gaitpost.2021.02.032 33677176

[35] Leardini A, Biagi F, Merlo A, Belvedere C, Benedetti MG. Multi-segment trunk kinematics during locomotion and elementary exercises. Clin Biomech (Bristol). 2011;26(6):562–71. doi: 10.1016/j.clinbiomech.2011.01.015 21419535

[36] Wallard L, Dietrich G, Kerlirzin Y, Bredin J. Balance control in gait children with cerebral palsy. Gait Posture. 2014;40(1):43–7. doi: 10.1016/j.gaitpost.2014.02.009 24656683

[37] Katz-Leurer M, Rotem H, Keren O, Meyer S. Balance abilities and gait characteristics in post-traumatic brain injury, cerebral palsy and typically developed children. Dev Neurorehabil. 2009;12(2):100–5. doi: 10.1080/17518420902800928 19340662

[38] Kim CJ, Son SM. Comparison of spatiotemporal gait parameters between children with normal development and children with diplegic cerebral palsy. J Phys Ther Sci. 2014;26(9):1317–9. doi: 10.1589/jpts.26.1317 25276007 PMC4175228

[39] Saether R, Helbostad JL, Adde L, Brændvik S, Lydersen S, Vik T. Gait characteristics in children and adolescents with cerebral palsy assessed with a trunk-worn accelerometer. Res Dev Disabil. 2014;35(7):1773–81. doi: 10.1016/j.ridd.2014.02.011 24679701

[40] Summa A, Vannozzi G, Bergamini E, Iosa M, Morelli D, Cappozzo A. Multilevel upper body movement control during gait in children with cerebral palsy. PLOS ONE. 2016;11:e0151792. doi: 10.1371/journal.pone.0151792 PMC4801392 26999362

[41] Heyrman L, Feys H, Molenaers G, Jaspers E, Monari D, Nieuwenhuys A, et al. Altered trunk movements during gait in children with spastic diplegia: compensatory or underlying trunk control deficit? Res Dev Disabil. 2014;35(9):2044–52. doi: 10.1016/j.ridd.2014.04.031 24864057

[42] Sæther R, Helbostad JL, Adde L, Braendvik S, Lydersen S, Vik T. The relationship between trunk control in sitting and during gait in children and adolescents with cerebral palsy. Dev Med Child Neurol. 2015;57(4):344–50. doi: 10.1111/dmcn.12628 25412837

[43] Balzer J, Marsico P, Mitteregger E, van der Linden ML, Mercer TH, van Hedel HJA. Influence of trunk control and lower extremity impairments on gait capacity in children with cerebral palsy. Disabil Rehabil. 2018;40(26):3164–70. doi: 10.1080/09638288.2017.1380719 28944697

[44] Armand S, Watelain E, Mercier M, Lensel G, Lepoutre F-X. Identification and classification of toe-walkers based on ankle kinematics, using a data-mining method. Gait Posture. 2006;23(2):240–8. doi: 10.1016/j.gaitpost.2005.02.007 16399521

[45] Worthen-Chaudhari L, Bing J, Schmiedeler JP, Basso DM. A new look at an old problem: defining weight acceptance in human walking. Gait Posture. 2014;39(1):588–92. doi: 10.1016/j.gaitpost.2013.09.012 24139684

[46] Beyaert C, Pierret J, Vasa R, Paysant J, Caudron S. Toe walking in children with cerebral palsy: a possible functional role for the plantar flexors. J Neurophysiol. 2020;124(4):1257–69. doi: 10.1152/jn.00717.2019 32877265

[47] Willerslev-Olsen M, Andersen JB, Sinkjaer T, Nielsen JB. Sensory feedback to ankle plantar flexors is not exaggerated during gait in spastic hemiplegic children with cerebral palsy. J Neurophysiol. 2014;111(4):746–54. doi: 10.1152/jn.00372.2013 24225545

[48] Lorentzen J, Willerslev-Olsen M, Hüche Larsen H, Farmer SF, Nielsen JB. Maturation of feedforward toe walking motor program is impaired in children with cerebral palsy. Brain. 2019;142(3):526–41. doi: 10.1093/brain/awz002 30726881

[49] Lorentzen J, Willerslev-Olsen M, Hüche Larsen H, Svane C, Forman C, Frisk R, et al. Feedforward neural control of toe walking in humans. J Physiol. 2018;596(11):2159–72. doi: 10.1113/JP275539 29572934 PMC5983220

[50] Honeine J-L, Schieppati M, Gagey O, Do M-C. The functional role of the triceps surae muscle during human locomotion. PLoS One. 2013;8(1):e52943. doi: 10.1371/journal.pone.0052943 23341916 PMC3547017

[51] Neptune RR, Kautz SA, Zajac FE. Contributions of the individual ankle plantar flexors to support, forward progression and swing initiation during walking. J Biomech. 2001;34(11):1387–98. doi: 10.1016/s0021-9290(01)00105-1 11672713

[52] Correa TA, Schache AG, Graham HK, Baker R, Thomason P, Pandy MG. Potential of lower-limb muscles to accelerate the body during cerebral palsy gait. Gait Posture. 2012;36(2):194–200. doi: 10.1016/j.gaitpost.2012.02.014 22522045

[53] Smith LR, Lee KS, Ward SR, Chambers HG, Lieber RL. Hamstring contractures in children with spastic cerebral palsy result from a stiffer extracellular matrix and increased in vivo sarcomere length. J Physiol. 2011;589(Pt 10):2625–39. doi: 10.1113/jphysiol.2010.203364 21486759 PMC3115830

[54] Pierret J, Beyaert C, Vasa R, Rumilly E, Paysant J, Caudron S. Rehabilitation of postural control and gait in children with cerebral palsy: the beneficial effects of trunk-focused postural activities. Dev Neurorehabil. 2023;26(3):180–92. doi: 10.1080/17518423.2023.2193269 36959769

[55] Santamaria V, Ai X, Chin K, Dutkowsky JP, Gordon AM, Agrawal SK. Study protocol for a randomised controlled trial to determine the efficacy of an intensive seated postural intervention delivered with robotic and rigid trunk support systems. BMJ Open. 2023;13(8):e073166. doi: 10.1136/bmjopen-2023-073166 37591642 PMC10441060

[56] Munaf A, Mehboob S, Razzaq M, Younas M, Umair S, Waseem I, et al. Effect of Trunk Exercises on Trunk Control, Balance, and Mobility Function in Children with Hemiparetic CP. Pak J Med Health Sci. 2022;16(11):95–8. doi: 10.53350/pjmhs2022161195

[57] Abidin N, Ünlü Akyüz E, Cankurtaran D, Karaahmet ÖZ, Tezel N. The effect of robotic rehabilitation on posture and trunk control in non-ambulatory cerebral palsy. Assist Technol. 2024;36(6):422–8. doi: 10.1080/10400435.2022.2059592 35385378

[58] Read HS, Hazlewood ME, Hillman SJ, Prescott RJ, Robb JE. Edinburgh visual gait score for use in cerebral palsy. J Pediatr Orthop. 2003;23(3):296–301. doi: 10.1097/01241398-200305000-00005 12724590

[59] Russell DJ, Avery LM, Walter SD, Hanna SE, Bartlett DJ, Rosenbaum PL, et al. Development and validation of item sets to improve efficiency of administration of the 66-item Gross Motor Function Measure in children with cerebral palsy. Dev Med Child Neurol. 2010;52(2):e48-54. doi: 10.1111/j.1469-8749.2009.03481.x 19811516

[60] McCoy SW, Bartlett DJ, Yocum A, Jeffries L, Fiss AL, Chiarello L, et al. Development and validity of the early clinical assessment of balance for young children with cerebral palsy. Dev Neurorehabil. 2014;17(6):375–83. doi: 10.3109/17518423.2013.827755 24087912

[61] Ma Y, Aslam R, Jester A. Development and validation of a WHO ICF compliant hand and upper limb assessment tool for children: The Reach Out questionnaire. J Hand Ther. 2023;36(4):1000–6. doi: 10.1016/j.jht.2023.03.005 37580195

[62] Chan A-W, Tetzlaff JM, Altman DG, Laupacis A, Gøtzsche PC, Krleža-Jerić K, et al. SPIRIT 2013 statement: defining standard protocol items for clinical trials. Ann Intern Med. 2013;158(3):200–7. doi: 10.7326/0003-4819-158-3-201302050-00583 23295957 PMC5114123

[63] Palisano R, Rosenbaum P, Walter S, Russell D, Wood E, Galuppi B. Development and reliability of a system to classify gross motor function in children with cerebral palsy. Dev Med Child Neurol. 1997;39(4):214–23. doi: 10.1111/j.1469-8749.1997.tb07414.x 9183258

[64] Yam WKL, Leung MSM. Interrater reliability of Modified Ashworth Scale and Modified Tardieu Scale in children with spastic cerebral palsy. J Child Neurol. 2006;21(12):1031–5. doi: 10.1177/7010.2006.00222 17156693

[65] McDonough AL, Batavia M, Chen FC, Kwon S, Ziai J. The validity and reliability of the GAITRite system’s measurements: A preliminary evaluation. Arch Phys Med Rehabil. 2001;82(3):419–25. doi: 10.1053/apmr.2001.19778 11245768

[66] Gouelle A. Use of functional ambulation performance score as measurement of gait ability: review. J Rehabil Res Dev. 2014;51(5):665–74. doi: 10.1682/JRRD.2013.09.0198 25333744

[67] Gouelle A, Rennie L, Clark DJ, Mégrot F, Balasubramanian CK. Addressing limitations of the Gait Variability Index to enhance its applicability: The enhanced GVI (EGVI). PLoS One. 2018;13(6):e0198267. doi: 10.1371/journal.pone.0198267 29856818 PMC5983480

[68] Joanna M, Magdalena S, Katarzyna B-M, Daniel S, Ewa L-D. The Utility of Gait Deviation Index (GDI) and Gait Variability Index (GVI) in detecting gait changes in spastic hemiplegic cerebral palsy children using Ankle-Foot Orthoses (AFO). Children (Basel). 2020;7(10):149. doi: 10.3390/children7100149 32992683 PMC7600809

[69] Prosser LA, Aguirre MO, Zhao S, Bogen DK, Pierce SR, Nilan KA, et al. Infants at risk for physical disability may be identified by measures of postural control in supine. Pediatr Res. 2022;91(5):1215–21. doi: 10.1038/s41390-021-01617-0 34175891 PMC8710181

[70] Kurz MJ, Arpin DJ, Corr B. Differences in the dynamic gait stability of children with cerebral palsy and typically developing children. Gait Posture. 2012;36(3):600–4. doi: 10.1016/j.gaitpost.2012.05.029 22743027

[71] Russell DJ, Avery LM, Rosenbaum PL, Raina PS, Walter SD, Palisano RJ. Improved scaling of the gross motor function measure for children with cerebral palsy: evidence of reliability and validity. Phys Ther. 2000;80(9):873–85. doi: 10.1093/ptj/80.9.873 10960935

[72] Avery LM, Russell DJ, Raina PS, Walter SD, Rosenbaum PL. Rasch analysis of the gross motor function measure: validating the assumptions of the Rasch model to create an interval-level measure. Arch Phys Med Rehabil. 2003;84(5):697–705. doi: 10.1016/s0003-9993(02)04896-7 12736885

[73] LaForme Fiss A, McCoy SW, Bartlett D, Avery L, Hanna SE, On Track Study Team. Developmental trajectories for the early clinical assessment of balance by gross motor function classification system level for children with cerebral palsy. Phys Ther. 2019;99(2):217–28. doi: 10.1093/ptj/pzy132 30715490 PMC6339982

[74] Randall KE, Bartlett DJ, McCoy SW. Measuring postural stability in young children with cerebral palsy: a comparison of 2 instruments. Pediatr Phys Ther. 2014;26(3):332–7. doi: 10.1097/PEP.0000000000000062 24979089

[75] Rygelová M, Uchytil J, Torres IE, Janura M. Comparison of spatiotemporal gait parameters and their variability in typically developing children aged 2, 3, and 6 years. PLoS One. 2023;18(5):e0285558. doi: 10.1371/journal.pone.0285558 37167236 PMC10174554

[76] Dewar R, Love S, Johnston LM. Exercise interventions improve postural control in children with cerebral palsy: a systematic review. Dev Med Child Neurol. 2015;57(6):504–20. doi: 10.1111/dmcn.12660 25523410

[77] Lee B-H. Relationship between gross motor function and the function, activity and participation components of the International Classification of Functioning in children with spastic cerebral palsy. J Phys Ther Sci. 2017;29(10):1732–6. doi: 10.1589/jpts.29.1732 29184279 PMC5684000

[78] Curtis DJ, Woollacott M, Bencke J, Lauridsen HB, Saavedra S, Bandholm T, et al. The functional effect of segmental trunk and head control training in moderate-to-severe cerebral palsy: a randomized controlled trial. Dev Neurorehabil. 2018;21(2):91–100. doi: 10.1080/17518423.2016.1265603 28045553

[79] Talgeri AJ, Nayak A, Karnad SD, Jain P, Tedla JS, Reddy RS, et al. Effect of trunk targeted interventions on functional outcomes in children with cerebral palsy- a systematic review. Dev Neurorehabil. 2023;26(3):193–205. doi: 10.1080/17518423.2023.2193265 37021364

[80] Lim M-S, Yoo B-C, Lim H-W. The effects of trunk intervention on gross motor function, balance, and spasticity in cerebral palsy: systematic review and meta-analysis. Medicina (Kaunas). 2025;61(8):1324. doi: 10.3390/medicina61081324 40870368 PMC12387430

[81] Beyaert C, Vasa R, Frykberg GE. Gait post-stroke: pathophysiology and rehabilitation strategies. Neurophysiol Clin. 2015;45(4–5):335–55. doi: 10.1016/j.neucli.2015.09.005 26547547

[82] Nielsen JB, Christensen MS, Farmer SF, Lorentzen J. Spastic movement disorder: should we forget hyperexcitable stretch reflexes and start talking about inappropriate prediction of sensory consequences of movement?. Exp Brain Res. 2020. doi: 10.1007/s00221-020-05792-0 32382862

[83] Abbes Z, Gomaa A-S, Al-Marri S, Haddad M. Relationship between physical characteristics and 200-metre swimming performance in young amateur swimmers. Acta kinesiol. 2023;17.(N1 2023). doi: 10.51371/issn.1840-2976.2023.17.1.1

