# Supplementary material for: Investigating rehabilitation by activities involving the trunk to improve balance and gait control in young children with cerebral palsy: A randomized open-label crossover trial protocol
Source: PLoS One. 2026 Mar 13;21(3):e0334195. doi: 10.1371/journal.pone.0334195 (PMC12987434; doi:10.1371/journal.pone.0334195)
Supplement: S1 File — (DOCX) [file pone.0334195.s001.docx]

**Rééducation par activités impliquant le tronc chez des jeunes enfants ayant une paralysie cérébrale**

| COMITE DE PROTECTION DES PERSONNES |  |
| --- | --- |
| Désignation | N° d’enregistrement |
|  | 2023-A01969-36 |
| ASSURANCE |  |
| HDI Global SE  Tour Trinity, 1 bis place de la Défense  CS 20298  92035 La Défense, CEDEX | N° de contrat : 0100534514058 240024 |

**Catégorie de la Recherche**

RIPH 1  RIPH 2  RIPH 3

**Promoteur :**

Nom : Institut Régional de Médecine Physique et de Réadaptation Louis PIERQUIN, affilié au laboratoire de recherche DEVAH EA3450 (développement, adaptation, handicap) de l’université de Lorraine

Adresse : 75, boulevard Lobau, CS 34209, 54042 Nancy Cedex, France

Tel : 03 83 52 97 00

Mél : cellule.recherche.ug-ne@ugecam.assurance-maladie.fr

**Signatures**

Promoteur Investigateur principal

**Promoteur :**

Nom : Institut Régional de Médecine Physique et de Réadaptation Louis PIERQUIN, affilié au laboratoire de recherche DEVAH EA3450 (développement, adaptation, handicap) de l’université de Lorraine

Adresse : 75, boulevard Lobau, CS 34209, 54042 Nancy Cedex, France

Tel : 03 83 52 97 00

Mél : cellule.recherche.ug-ne@ugecam.assurance-maladie.fr

**Demandeur :**

*Personne autorisée par le promoteur à déposer le dossier au Comité de Protection des Personnes*

Prénom, Nom : Jonathan Pierret

Titre : Docteur en Sciences de la Vie et de la Santé

Fonction : Responsable Cellule Recherche Clinique et Innovation

Adresse : 75, boulevard Lobau, CS 34209, 54042 Nancy Cedex, France

Tel : 03 83 52 67 61

Mél : cellule.recherche.ug-ne@ugecam.assurance-maladie.fr

**Investigateur Coordonnateur:**

Prénom, Nom : Christian Beyaert

Qualité : PU-PH Physiologie, Pédiatre

N°RPPS : 100022353976

Adresse : CMPRE, 46 rue du Doyen Parisot – CS 20002, 54630 Flavigny-sur-Moselle

Tel secrétariat : 0354591923

Mél : christian.beyaert@univ-lorraine.fr

**Autres investigateurs, collaborateurs et scientifiques**

L’annexe 1 présente les investigateurs, collaborateurs médecins et scientifiques impliqués dans ce protocole de recherche.

**Site d’investigation clinique**

L’annexe 2 présente les sites d’investigation clinique

**Comité scientifique :**

Le comité scientifique est composé des personnes suivantes :

- Investigateur coordonnateur : Pr Christian Beyaert
- Investigateur(s) spécialiste(s) de la pathologie et des thérapeutiques étudiées : Dr Fanny Dalmont
- Représentant du promoteur : Jonathan Pierret
- Méthodologiste(s) : Christelle Requena
- Collecte et traitement de données : Stella Zografou

**Historique des modifications du protocole :**

| **Version** | **Date** | **Description des modifications** |
| --- | --- | --- |
|  |  |  |
|  |  |  |

Liste des abréviations

ANSM : Agence Nationale de Sécurité du Médicament

CNIL : Commission Nationale Informatique et Liberté

CPP : Comité de Protection des Personnes

IRR : Institut Régional de Réadaptation

MPR : Médecin en Médecine Physique et de Réadaptation

PMSI : Programme de Médicalisation des Systèmes d’Information

RIPH : Recherche Impliquant la Personne Humaine

Tables des matières

Contenu

[Liste des abréviations 5](#_Toc158717453)

[Tables des matières 6](#_Toc158717454)

[Protocole ActivTronc 8](#_Toc158717455)

[1. Contexte scientifique 8](#_Toc158717456)

[2. Objectif(s), critère(s) de jugement et hypothèse(s) de l’étude 12](#_Toc158717457)

[2.1. Objectif principal et hypothèse 12](#_Toc158717458)

[2.2. Critère de jugement principal 12](#_Toc158717459)

[2.3. Objectif(s) secondaire(s) et hypothèses 12](#_Toc158717460)

[2.4. Critères de jugement secondaires 13](#_Toc158717461)

[3. Méthodologie 13](#_Toc158717462)

[3.1. Type d’étude 13](#_Toc158717463)

[3.2. Population étudiée 13](#_Toc158717464)

[3.2.1. Critères d’inclusion 14](#_Toc158717465)

[3.2.2. Critère de non-inclusion 14](#_Toc158717466)

[3.2.3. Calcul d’effectif 14](#_Toc158717467)

[3.3. Randomisation 15](#_Toc158717468)

[3.4. Plan expérimental 15](#_Toc158717469)

[4. Déroulement de la Recherche 16](#_Toc158717470)

[4.1. Procédure de recrutement des enfants 16](#_Toc158717471)

[4.2. Contenus de rééducation 18](#_Toc158717472)

[4.3. Description des évaluations réalisées au cours de la recherche 19](#_Toc158717473)

[4.3.1. Evaluations cliniques 19](#_Toc158717474)

[4.3.2. Analyse instrumentale de la marche et de la station debout 20](#_Toc158717475)

[4.4. Résultats attendus 22](#_Toc158717476)

[4.5. Plan d’analyse statistique 22](#_Toc158717477)

[4.6. Sorties d’étude et critère d’arrêt de l’étude 23](#_Toc158717478)

[4.7. Durée de la recherche et indemnisation 23](#_Toc158717479)

[4.8. Période d’exclusion et exclusivité 24](#_Toc158717480)

[5. Bénéfices et risques 24](#_Toc158717481)

[5.1. Bénéfices pour le participant 24](#_Toc158717482)

[5.2. Risques et contraintes pour le participant 24](#_Toc158717483)

[6. Evaluation de la sécurité 25](#_Toc158717484)

[6.1. Evènements indésirables 25](#_Toc158717485)

[6.1.1. Définitions 25](#_Toc158717486)

[6.1.2. Evènement(s) indésirable(s) grave(s) 25](#_Toc158717487)

[6.2. Conduite à tenir 26](#_Toc158717488)

[6.2.1. Conduites à tenir en cas d’évènement indésirable grave 27](#_Toc158717489)

[7. Gestion des données 28](#_Toc158717490)

[7.1. Droit d’accès aux données 28](#_Toc158717491)

[7.2. Données sources 28](#_Toc158717492)

[7.3. Anonymats des participants 29](#_Toc158717493)

[7.4. Confidentialité 29](#_Toc158717494)

[7.5. Archivage 29](#_Toc158717495)

[7.6. Règles relatives à la publication au rapport final 29](#_Toc158717496)

[8. Assurance Qualité 29](#_Toc158717497)

[8.1. Engagement de l’investigateur - Bonnes pratiques cliniques 29](#_Toc158717498)

[8.2. Contrôle de la qualité des données 30](#_Toc158717499)

[8.3. Audit et Inspection 30](#_Toc158717500)

[9. Organisation de l’étude 30](#_Toc158717501)

[9.1. Comité scientifique 30](#_Toc158717502)

[10. Considérations éthiques et règlementaires 30](#_Toc158717503)

[10.1. Autorisation de lieu de recherche 31](#_Toc158717504)

[10.2. Conduite éthique de l’étude 31](#_Toc158717505)

[10.3. Comité de Protection des Personnes 31](#_Toc158717506)

[10.4. Responsabilités de l’investigateur 31](#_Toc158717507)

[10.5. Responsabilités du promoteur 32](#_Toc158717508)

[10.6. Déclaration des fichiers 32](#_Toc158717509)

[11. Bibliographie 33](#_Toc158717510)

[12. Annexes 37](#_Toc158717511)

[12.1. Annexe 1 : liste des investigateurs et des collaborateurs scientifiques 37](#_Toc158717512)

[12.2. Annexe 2 : liste des sites d’investigation clinique 39](#_Toc158717513)

Protocole ActivTronc

1. Contexte scientifique

La paralysie cérébrale (PC) décrit un groupe de troubles moteurs et posturaux permanents, limitant l'activité, causés par des lésions du cerveau en développement in utero ou post-partum. Les réorganisations ultérieures des fonctions du système nerveux central chez les enfants atteints de PC entraînent également des troubles moteurs (1). Le développement typique du contrôle segmentaire du tronc, qui se produit progressivement au cours de l'enfance (2), est étroitement lié aux performances de la motricité globale chez les jeunes nourrissons (3) et est considérablement retardé chez les prématurés (4). Dès la petite enfance, les enfants atteints de PC présentent des troubles du contrôle axial et un contrôle postural anormal en position assise (5). Nous avons récemment montré que les enfants atteints de PC présentaient des déficiences spécifiques dans le contrôle du tronc lors d'une tâche d’auto-stabilisation sur un dispositif d'assise instable par rapport aux enfants au développement typique (TD) (6). Ces déficiences dans le contrôle postural axial (et en particulier dans le contrôle du tronc) restent présentes tout au long de la période de développement moteur - même après que l'enfant puisse se tenir debout et marcher de manière indépendante (7).

Pendant la marche, les enfants ayant une PC présentent des déviations significatives de la cinématique et de la cinétique du tronc ; les amplitudes de mouvement du tronc dans les trois plans de l'espace sont beaucoup plus grandes que chez les enfants ayant un TD (8). Ces déviations sont associées à un mauvais contrôle de l'équilibre dynamique pendant la marche (9), ce qui se traduit par des largeurs de pas plus importantes (10), une plus grande variabilité de la longueur des pas, des accélérations plus importantes de la tête, du thorax (tronc supérieur), du bas du dos (région L3), du bassin et du centre de masse du corps dans les trois plans sur l'ensemble du cycle de marche et une plus grande instabilité du bas du dos par rapport aux enfants TD (11,12). Étant donné que le tronc et les membres inférieurs interagissent réciproquement pendant la marche, chez les enfants ayant une PC, les déviations du tronc entraînent des déviations des membres inférieurs, et vice versa (7,13).

La marche digitigrade est l'une des déviations des membres inférieurs les plus courantes chez les enfants atteints de PC. Elle se définit comme l'absence du premier pivot (sur le talon) lorsque les enfants touchent le sol avec le pied à plat ou avec l'avant-pied lors du contact initial (CI) (14). Ainsi, le CI est immédiatement suivi du deuxième pivot, et le tibia roule sur la cheville. Au CI, l'angle de la cheville est souvent en flexion plantaire, c'est-à-dire en équin. L'absence du premier pivot chez les enfants atteints de PC est également associée à une absorption intense et précoce de l'énergie et à un travail négatif exercé au niveau de l'articulation de la cheville (15), ce qui a pour effet de décélérer la dorsiflexion de la cheville et l'inclinaison antérieure du tibia pendant la phase de mise en charge (MC) de la marche (définie comme la période initiale d'absorption combinée d’énergie au niveau des articulations des membres inférieurs) (16). Ce comportement implique une activité précoce du triceps sural, qui commence en fin d'oscillation et dure pendant toute la phase de MC (17). On considère généralement que l'activité prolongée des fléchisseurs plantaires pendant la marche chez les enfants atteints de PC est due à la spasticité (réflexes d'étirement hyperexcitables) et qu'elle induit un équin (1). Néanmoins, l'existence et/ou la signification fonctionnelle des réflexes d'étirement exagérés pendant la marche chez les patients spastiques ont fait l'objet de débats pendant des décennies (18). En particulier, il est peu probable que la spasticité contribue à la marche digitigrade chez les enfants ayant une PC ; l'activité du soléaire pendant la phase d'oscillation est réduite (15), et une activité réflexe exagérée est absente (19). En revanche, la marche digitigrade chez les enfants ayant une PC et chez les enfants ayant un développement typique (DT) est caractérisée par un contrôle en anticipation des muscles de la cheville - ce qui suggère que ce modèle de marche fait partie d'un processus adaptatif (20).

Lorsque le deuxième pivot commence, les fléchisseurs plantaires décélèrent la dorsiflexion de la cheville, ralentissent la progression du tronc vers l'avant et soutiennent le corps en l'accélérant vers le haut (21). Cette accélération du corps vers le haut peut être réalisée soit en déplaçant le centre de masse (CdM) vers le haut, soit en décélérant le mouvement du CdM vers le bas. Chez les enfants ayant une PC, l'activité des fléchisseurs plantaires associée au deuxième pivot précoce en MC entraîne une puissance négative de la cheville, ce qui ralentit la dorsiflexion de la cheville et décélère ainsi le CoM du corps vers le bas et vers l'avant (22). Toutes ces actions contribuent au travail négatif plus important exercé sur le CdM par la jambe antérieure chez les enfants ayant une PC, par rapport aux enfants ayant un TD (23). La présence des troubles du contrôle du tronc mentionnés ci-dessus et d'une altération du contrôle de l'équilibre dynamique pendant la marche chez les enfants ayant une PC suggère que l'activation précoce des fléchisseurs plantaires et la forte puissance négative de la cheville pendant la MC associées à la marche digitigrade pourraient correspondre à un mécanisme adaptatif pour décélérer les déplacements du tronc vers l'avant et vers le bas, afin de compenser un mauvais équilibre et un mauvais contrôle postural du tronc.

Chez des enfants ayant une paralysie cérébrale (PC), âgés de 5 à 12 ans, avec une altération du contrôle du tronc et une marche autonome digitigrade, nous avons récemment montré, comparés aux enfants ayant un DT, une largeur de pas plus grande (en raison d'une altération de l'équilibre) (10), un pic plus important de décélération antérieure du sternum, de décélération vers le bas du sacrum et de puissance négative de la cheville pendant la phase de MC (24) (voir Figure 1).


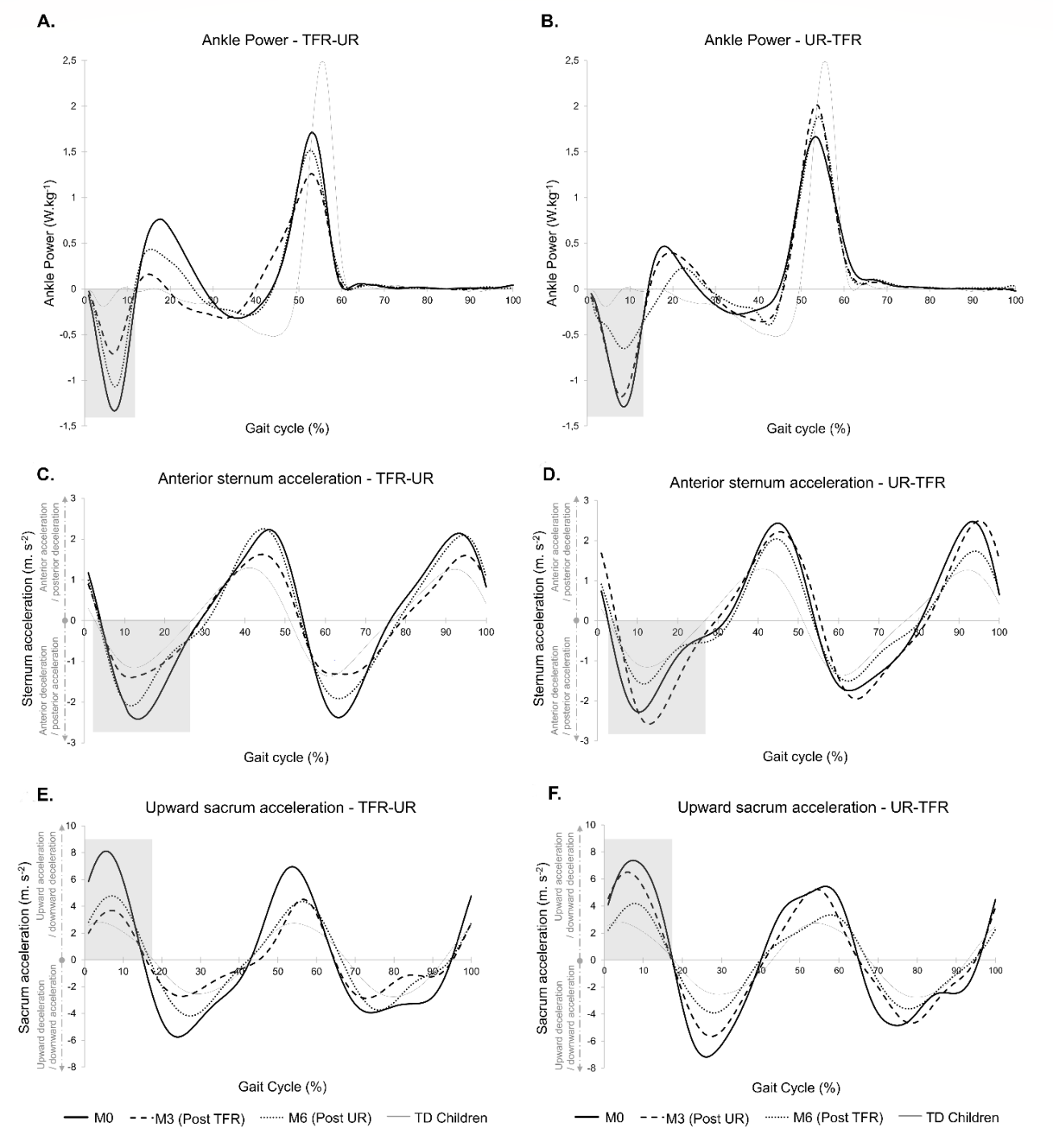


Figure 1 : Puissance totale moyenne de la cheville (A, B), accélération antéropostérieure moyenne du marqueur du sternum (C, D) et accélération ascendante moyenne (c'est-à-dire décélération descendante) du marqueur du sacrum (E, F), pendant la marche, chez des enfants ayant une paralysie cérébrale (PC) qui ont effectué une TFR puis une UR (A, C, E, n=8) ou une UR puis une TFR (B, D, F, n=9), et chez des enfants au DT (tous les panneaux, n=17). TFR : rééducation centrée sur le tronc (=RAIT) ; UR : rééducation habituelle (=RH) ; TD : développement typique. Les lignes noires correspondent au groupe PC (ligne pleine pour M0, ligne pointillée pour M3 et ligne en pointillé pour M6) et la ligne grise pleine correspond au groupe DT à M0. Le rectangle correspond à la zone d'intérêt pour le pic pendant la phase d'acceptation du poids.

Le pic de décélération du sternum et du sacrum étaient significativement corrélés avec le pic de puissance négative de la cheville pendant la phase de MC, ce qui est compatible avec l'action des fléchisseurs plantaires sur le tronc ou le CdM (22,25). Cette étude, en cross over, a permis aussi d’évaluer l’effet d’une rééducation basée sur des activités impliquant fortement le tronc (RAIT) pendant 3 mois par rapport à une rééducation habituelle (RH) reposant principalement sur une rééducation conventionnelle basée sur le traitement de groupes musculaires (étirement, renforcement, réduction du tonus) des membres inférieurs. Seule la RAIT a amélioré significativement le contrôle postural du tronc, mis en évidence par l’amélioration du score de l'échelle de mesure du contrôle du tronc (26) et de l’auto stabilisation posturale sur un dispositif d’assise instable (6). De plus, seule la RAIT a réduit significativement les pics de décélération sternale et sacrée et la puissance négative couplée de la cheville en phase de MC (voir Figure 1). Il est ainsi fortement suggéré que les fléchisseurs plantaires compensent l'altération du contrôle postural du tronc - une cible clé de la rééducation chez les enfants ayant une PC.

Il existe donc un fort intérêt à rééduquer le tronc chez les enfants atteints de PC. En effet, si notre étude montre des bénéfices sur des paramètres liés à l’équilibre dynamique au cours de la marche, de nombreuses autres études ont fait du tronc la cible principale de leur intervention (27–32). Ces études montrent notamment une amélioration du contrôle du tronc, mais également de la marche (paramètres spatio-temporels, performance lors du Timed-Up-and-Go, du test de marche de 1 minute et du test de marche de 6 minutes) ainsi que la fonction motrice globale. Par ailleurs, les études ayant testés des protocoles d’équithérapie chez les enfants atteints de PC mettent en lien les améliorations observées sur l’équilibre, la marche et la fonction motrice globale, avec les améliorations induites sur le contrôle du tronc (33–36).

Du fait de l’importance du contrôle du tronc sur la fonction motrice globale, l’équilibre et la marche des enfants atteints de PC, et au vu de l’ensemble des études montrant le bénéfice d’une rééducation centrée sur le tronc, des protocoles de rééducation centrés sur le tronc sont à présent prescrits aux patients de notre centre dont le profil semble indiquer un intérêt pour un renforcement du tronc.

L’étude de l’effet d’une RAIT sur la capacité de stabilisation en position assise instable et sur la dynamique du tronc et de la cheville au cours de la marche, que nous avons mené, a été réalisée chez des enfants atteints d’une PC âgés de 5 à 12 ans en partie en raison de limitations techniques liées à l’assise instable et à l’analyse tridimensionnelle du mouvement avec de nombreux marqueurs à placer sur la peau, difficilement réalisables pour des enfants de moins de 5 ans. Cependant, il apparait pertinent de réaliser précocement, dès l’âge de 18 mois, une rééducation comme la RAIT en vue d’améliorer le contrôle postural de l’équilibre et la dynamique de marche chez les enfants atteints de PC. Dans cette optique et afin de simplifier l’exploration fonctionnelle de la dynamique de marche, et de pouvoir évaluer les effets de la RAIT prescrite à nos patients les plus jeunes, nous avons acquis des centrales inertielles pour mesurer la décélération du sternum et celle du sacrum (L5) et un tapis de marche à capteurs de pression relative (figure 2) pour analyser l’instabilité de la marche via les paramètres spatio-temporels et mesurer, en début d’appui, l’importance du déplacement précoce vers l’avant de l’appui plantaire lié à la marche de type digitigrade. L’analyse du contrôle postural de la station debout est aussi réalisée de façon simplifiée grâce à l’utilisation d’une centrale inertielle placée en L5 estimant les déplacements du centre de masse corporel (logiciel mSway). Des scores cliniques sont aussi réalisés pour une évaluation de l’équilibre et de la motricité fonctionnelle globale.

Par ailleurs, la RAIT présente d’autres bénéfices potentiels tels qu’une amélioration de l’utilisation fonctionnelle du membre supérieur et de la main, observée en marge de l’étude chez les enfants de 5 à 12 ans (24). Aussi, dans cette étude nous explorons la fonction motrice du membre supérieur et de la main via un questionnaire adressé aux parents.


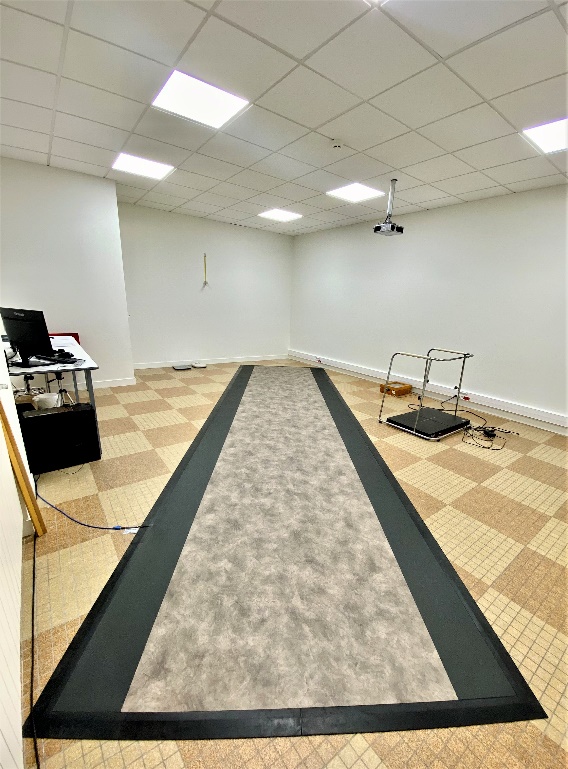


Figure 2 : Piste de marche munie de capteurs de pression relative (Zeno ®) permettant une analyse étendue et simplifiée des paramètres spatio-temporels et de la dynamique des empreintes plantaires.

1. Objectif(s), critère(s) de jugement et hypothèse(s) de l’étude
   1. Objectif principal et hypothèse

L’objectif primaire de l’étude est de montrer que le protocole de RAIT, prescrit sur une période de 3 mois, et non une RH, permet de réduire le pic de décélération vers l’avant du sternum en début d’appui (les 25 premiers % du cycle de marche) chez des enfants atteints de PC âgé de 18 mois à 5 ans et 6 mois et ayant une marche autonome ou avec utilisation inconstante d’une aide à la marche (déambulateur, béquilles, etc.), ce qui correspond aux niveaux I à II du système de classification de la fonction motrice globale (GMFCS) (37). Les enfants utilisant de façon inconstante une aide de marche devront être capables de marcher de façon autonome sur une distance d’environ 10 m afin de permettre l’évaluation sur la piste de marche.

- 1. Critère de jugement principal

Pic de décélération vers l’avant du sternum pendant les 25 premiers % du cycle de marche.

- 1. Objectif(s) secondaire(s) et hypothèses

Les objectifs secondaires, pour les critères de jugement secondaires décrits au paragraphe 2.4 ci-dessous, visent :

- (1) à montrer une altération de ces critères chez les enfants atteints de PC de l’étude par rapport à des enfants à développement typique (DT) ;
- (2) à montrer une amélioration de ces critères significativement supérieure après 3 mois de RAIT qu’après 3 mois de RH ;
- (3) à montrer une amélioration supplémentaire de ces critères lorsque la durée de RAIT est augmentée.
  1. Critères de jugement secondaires
- (1) Variables liées à la qualité de la marche spontanée :
  - Pic de décélération vers le bas de L5 pendant les 25 premiers % du cycle de marche
  - Pourcentage de charge sur la moitié antérieure du pied lors du premier double appui
  - Largeur de pas normalisée en fonction de la largeur de bassin
  - eGVI : index de variabilité de la marche (38,39)

(2) Variables liées au contrôle postural de la station debout :

- - Vitesse moyenne de déplacement horizontal du centre de masse corporel
  - Ellipse à 95% de la surface de déplacement horizontal du centre de masse corporel

(2) Variables fonctionnelles évaluées par des scores cliniques :

- - Score à l’Echelle Clinique Précoce de l’Equilibre (traduction française de l’*Early Clinical Assessment of Balance*) (40)
  - Score à l’Evaluation de la Fonction Motrice Globale 66 séries d’items (traduction française du GMFG 66 IS) (41)
  - Score visuel de marche d’Edinbourg (42)

1. Méthodologie
   1. Type d’étude

Etude interventionnelle monocentrique randomisée contrôlée.

- 1. Population étudiée

Enfants atteints de paralysie cérébrale présentant des troubles du schéma de marche vus en consultation externe et/ou pris en charge (hospitalisation de jour ou complète) au CMPRE de Flavigny-sur-Moselle. La paralysie cérébrale est une affection de longue durée faisant l’objet d’un suivi médical spécialisé bien codifié. En Lorraine, le suivi thérapeutique multidisciplinaire de cette population est coordonné par un réseau de médecins spécialisés en médecine physique et de réadaptation, en neuropédiatrie et en pédiatrie intervenant dans plusieurs établissements. Les enfants atteints de PC recrutés pour cette étude seront des enfants ayant une hémiparésie ou une paraparésie de type spastique, une marche autonome sans aide à la marche (GMFCS I à II). Dans le cas où l’enfant a une paraparésie, seul le membre inférieur avec le soléaire le plus spastique sera analysé. Le soléaire le plus spastique se définit comme celui pour lequel on observe la plus grande différence entre l’amplitude du mouvement passif lent de dorsiflexion et l’angle obtenu en utilisant la vitesse maximale de dorsiflexion V3, selon l’échelle modifiée de Tardieu (43).

- - 1. Critères d’inclusion

Pour les enfants atteints de PC

- Age entre 18 mois et 5 ans et 6 mois
- PC de type paraparésie spastique ou hémiparésie spastique, GMFCS I à II
- Pas de rétraction ou rétraction modérée du triceps sural (dorsiflexion de la cheville : > 5° lors de l’examen clinique, genou tendu)
- Niveau de compréhension suffisant pour réaliser les activités impliquant le tronc sous forme d’auto-exercices (protocole de rééducation) ainsi que les évaluations cliniques et les explorations fonctionnelles.
- Acceptation du kinésithérapeute en charge du suivi de l’enfant de collaborer dans la réalisation du RAIT
- Affilié(e) à un régime de sécurité sociale

Pour les enfants à DT

- Age entre 18 mois et 5 ans et 6 mois
- Marche acquise avant l’âge de 18 mois
- Niveau de compréhension suffisant pour réaliser les évaluations cliniques et les explorations fonctionnelles
- Affilié(e) à un régime de sécurité sociale
  - 1. Critère de non-inclusion

Pour les enfants atteints de PC

• Chirurgie préalable des membres inférieurs depuis moins de 1 an

• Injection de toxine botulique A depuis moins de 6 mois

• Toute modification de prise en charge rééducative et/ou orthopédique depuis moins de 2 mois

• Flessum de hanche > 20°

• Présence d’une douleur subaiguë ou chronique au cours de la station debout ou de la marche

Pour les enfants à DT

• Trouble neurologique et/ou orthopédique pouvant influencer la marche

- - 1. Calcul d’effectif

Le calcul du nombre de sujets nécessaires est réalisé sur le critère principal. La distribution du pic de décélération du sternum en début d’appui était de type gaussienne au cours de notre précédente étude. En moyenne cette distribution était centrée sur 3,0 ± 1,1 m.s-2 (moyenne ± 1 écart type) chez les enfants avec PC et sur 1,1 ± 0,3 m.s-2 chez les enfants à DT (24). L’effet escompté de la RAIT est une réduction minimale du pic de décélération du sternum en début d’appui de un tiers de sa valeur, à l’image de l’effet de la RAIT dans l’étude précédente (24). Afin de montrer un effet de la RAIT à l’aide d’une ANOVA à mesure répétées dans laquelle nous nous attendons à une taille d’effet faible, avec un seuil alpha fixée à 0,05 et une puissance de 80%, un effectif total de 24 patients, soit 12 par groupe, est nécessaire (calcul réalisé à l’aide du logiciel G*Power 3.1 ; figure 3). Nous nous attendons à une plus grande variabilité des variables dynamiques de marche (attendue chez des enfants de 18 mois à 5 ans et 6 mois par rapport à des enfants de 5 à 12 ans). De ce fait, nous envisageons le recrutement de 32 enfants PC (16 par groupe), pour faire face à cette variabilité plus importante et pour pallier d’éventuels perdus de vues.


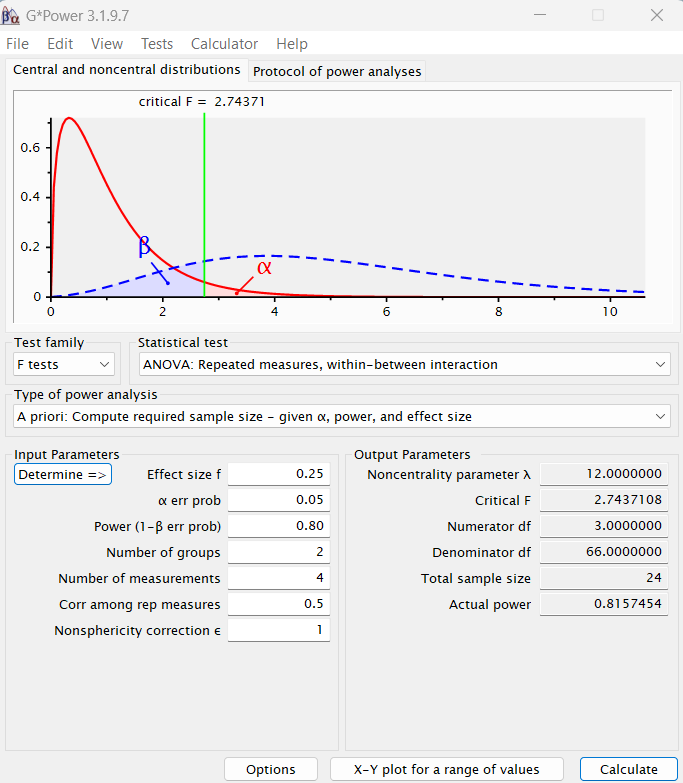


**Figure 3** : calcul du nombre de sujets nécessaires réalisé à l’aide du logiciel G*Power 3.1.

- 1. Randomisation

L’échantillon d’enfants atteints de PC sera divisé en deux groupes : PC1 et PC2, avec une affectation aléatoire individuelle des participants dans chacun des groupes par blocs de 4 ou 8 enfants. Cette affectation sera réalisée à l’aide d’un programme spécifique codé sur le logiciel Matlab.

Un échantillon témoin d’enfants à DT (n=32) sera apparié par rapport à l’âge et au sexe des enfants atteints de PC.

- 1. Plan expérimental

Le projet prévoit un examen clinique et des explorations fonctionnelles instrumentales à quatre périodes. Le plan expérimental est schématisé à la figure 4. Les évaluations sont décrites au chapitre « 4.3. Mesures – Description des évaluations réalisées au cours de la recherche ».

Les évaluations se feront à M0, M3 (M0 + 3 mois), M6 (M0 + 6 mois) et M12 (M0 + 12 mois) pour les enfants avec PC et à M0 pour les enfants à DT. Ainsi trois périodes de rééducation seront prévues : la première entre M0 et M3, la seconde entre M3 et M6 et la troisième entre M6 et M12.

Un premier groupe d’enfants (groupe PC1) poursuivra sa rééducation habituelle (RH) pendant les 3 premiers mois puis aura une RAIT les 9 mois suivants (PC1 = RH-RAIT). Le deuxième groupe d’enfants PC2 aura d’emblée la RAIT pendant les 12 mois de l’étude (PC2 = RAIT-RAIT). L’étude précédente réalisée chez des enfants avec PC de 5 à 12 ans ayant montré un effet thérapeutique uniquement lié à la RAIT (et non à la RH), la RAIT sera poursuivie chez les enfants en ayant bénéficié dès la première période de 3 mois et chez tous les enfants pour les 9 derniers mois de l’étude. Une telle planification permettra : 1) de faire bénéficier à tous les enfants avec PC de la RAIT ; 2) de mesurer l’effet, pendant 3 mois, de la RAIT comparé à celui de la RH ; 3) de tester la reproductibilité de l’effet de la RAIT pendant 3 mois entre les deux groupes et 4) de tester l’effet d’une RAIT supplémentaire de 3 mois dans le groupe PC2 (M6 versus M3) et de 6 mois dans les 2 groupes (M12 versus M6).


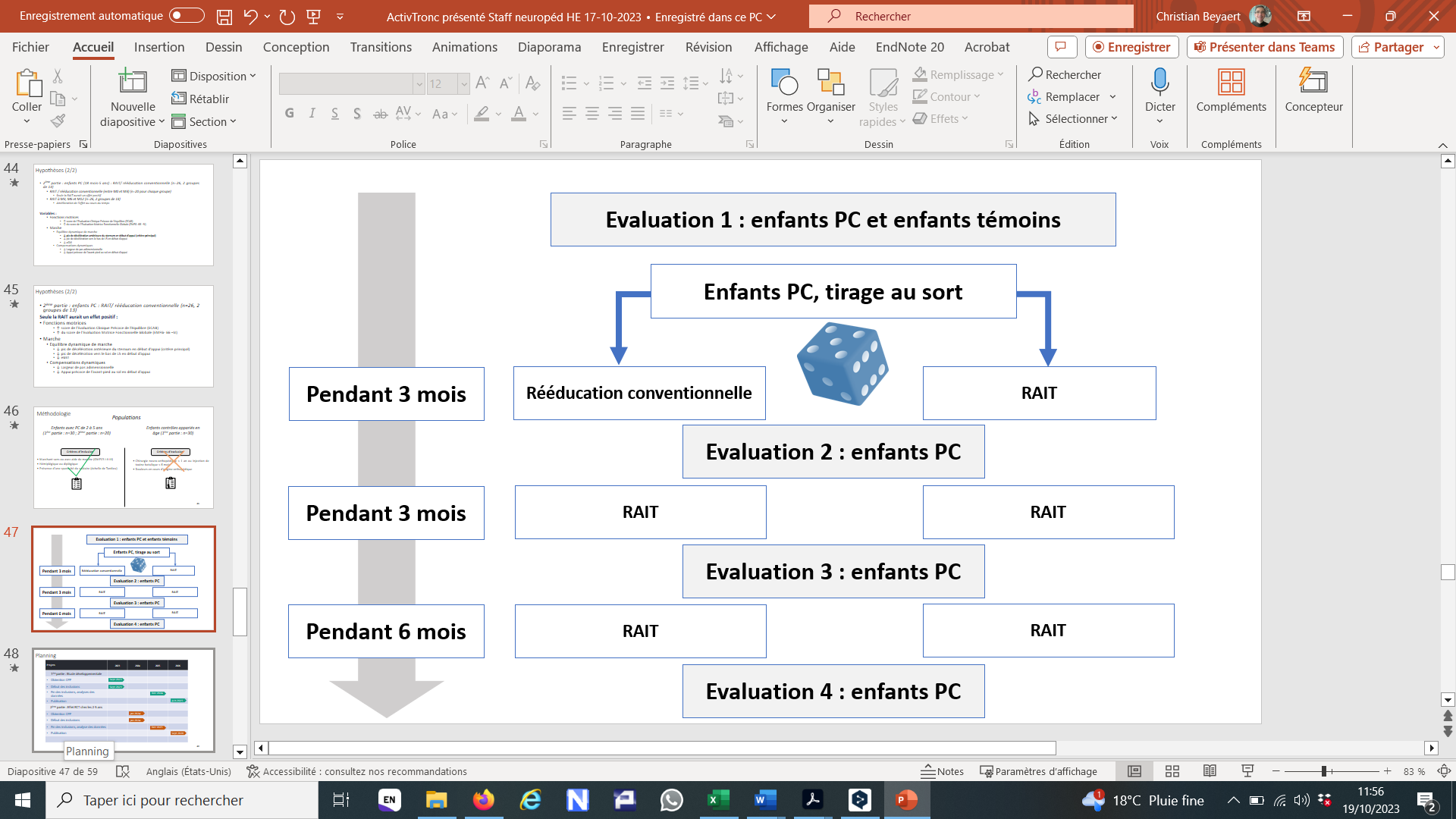


Figure 4 : plan expérimental de l'étude, RAIT : rééducation par activités posturales impliquant le tronc ; la rééducation conventionnelle repose sur le traitement de groupes musculaires (étirement, renforcement, réduction du tonus) des membres inférieurs.

1. Déroulement de la Recherche
   1. Procédure de recrutement des enfants

Le Centre de Médecine Physique et de Réadaptation de l’Enfance (CMPRE) à Flavigny sur Moselle, est un centre de rayonnement régional assurant diverses actions d’évaluation fonctionnelle et de traitement pour des enfants ayant des anomalies orthopédiques, neurologiques ou neuro-orthopédiques incluant des enfants ayant une PC. Des sessions d’évaluation motrice fonctionnelle y sont proposées et comprennent des évaluations cliniques (bilans neuro-orthopédiques et scores de motricité fonctionnelle) et des explorations fonctionnelles de la marche et de la station debout utilisant une piste de marche à capteurs de pression relatives, des caméras vidéo et des centrales inertielles.

Le diagnostic initial et le suivi médical des enfants ayant une PC sont réalisés par des médecins en interaction dans différents cadres : Réseau d’Accompagnement des Familles En Lorraine (RAFAEL), consultations de neuropédiatrie à l’Hôpital d’enfants de Brabois à Vandœuvre-lès-Nancy, Centres d’Action Médico-Sociale Précoce (CAMSP) de la Lorraine, consultations de pédiatrie en activité libérale ou consultations au Centre de Médecine Physique et de Réadaptation pour Enfants à Flavigny sur Moselle. La sollicitation de ces différents médecins à l’échelle de la Lorraine devrait permettre l’inclusion de 32 enfants avec PC sur une période de 30 mois.

Lorsqu’un de ces médecins, au cours d’une consultation avec un enfant ayant une PC, prescrit une évaluation motrice fonctionnelle associée à une consultation médicale réalisées au CMPRE et que cet enfant est potentiellement éligible pour l’étude, il en informe oralement les parents dans un échange interactif et leur confie la fiche d’information et de consentement en préparation d’une éventuelle inclusion dans l’étude, s’ils sont d’accord. Lors de la prise de rendez-vous au CMPRE, les références du kinésithérapeute qui prend en charge l’enfant sont renseignées afin qu’ il ou elle soit informé(e) de l’étude et que son consentement à y participer soit obtenu, en cas d’inclusion de l’enfant.

Lors de la consultation médicale associée à l’évaluation motrice fonctionnelle de l’enfant, l’un des médecins recruteurs du CMPRE (Dr Christian Beyaert, Dr Fanny Dalmont, Dr Marion Birck) explique aux parents et à l’enfant, selon son niveau de compréhension, l’étude sur la base des documents en leur possession au cours d’un échange interactif et leur propose d’y participer si les critères d’éligibilité sont respectés. S’ils sont d’accord, ils signent le formulaire de consentement préalablement confié, et l’inclusion de leur enfant dans le groupe avec RAIT initiale ou dans le groupe avec rééducation habituelle initiale est allouée selon une randomisation préalable. Si les parents désirent réfléchir davantage avant d’accepter ou de refuser la participation de leur enfant dans l’étude, un délai supplémentaire de 7 jours leur sera proposé.

Pour le groupe témoin d’enfants à DT, le recrutement se fera au moyen d’une annonce par courriel présentant l’étude et incluant la fiche d’information et de consentement destinée à des personnes de la Métropole du Grand Nancy, au sein du personnel de l’Université de Lorraine non-hiérarchiquement lié aux investigateurs de l’étude ou dont les enfants fréquentent des association sportives et éducatives. Les enfants à développement typique de la famille des enfants atteints de PC participant à l’étude seront également sollicités. De la même manière que les enfants atteints de PC, ils seront reçus en consultation avec un médecin investigateur.

Au cours de cette consultation, l’investigateur aura la responsabilité d’informer le participant et ses parents de la nature de l’étude, de ses objectifs, de sa méthodologie, de sa durée, des bénéfices attendus, des contraintes et risques prévisibles y compris en cas d'arrêt de l’étude avant son terme, des modalités de prise en charge médicale prévues en fin de recherche si nécessaire. Cette discussion informative sera basée sur la lettre d’information fournie préalablement aux parents. Les parents (et l’enfant participant(e), dans les limites liées à son jeune âge) auront toute latitude pour poser toute question concernant la recherche et seront informés de leur droit de refuser de participer à la recherche ou de retirer leur consentement à tout moment sans préjudice et sans avoir à justifier les raisons de leur décision.

Les enfants et leurs parents seront informés oralement dans un langage compréhensible du déroulement de l’étude et des examens à réaliser. Le formulaire de consentement éclairé sera signé en deux exemplaires par les différentes parties, après un temps supplémentaire de réflexion si les parents le désirent. L'investigateur et les parents de l’enfant qui se prête à la recherche conserveront chacun un original du formulaire de consentement.

L'information destinée aux parents et toute autre information écrite qui leur est donnée devront être actualisées si de nouvelles données sont disponibles, susceptibles de modifier leur avis ou si un amendement au protocole impose une correction de l'information et du formulaire de consentement. L'investigateur informera les parents de l’enfant de ces modifications et leur demandera de confirmer son accord en datant et signant un nouveau formulaire de consentement. Toute modification de ces documents doit être approuvée par le CPP.

- 1. Contenus de rééducation

Chaque enfant atteint de PC continuera à participer à une à trois séances de rééducation par semaine pendant un total de 12 mois (3 mois initialement de RH ou de RAIT puis 9 mois de RAIT) sous la direction du kinésithérapeute de l’enfant, dans la mesure où il ou elle accepte de participer à l’équipe et n’a pas participé à l’étude précédente utilisant le RAIT. Le kinésithérapeute est alors informé initialement des principes de la RAIT et reçoit des instructions détaillées sur son contenu peu avant le début de la période de traitement par RAIT. Pour les enfants qui sont pris en charge au CMPRE de Flavigny-sur-Moselle, c’est un kinésithérapeute du centre qui s’occupera de suivre la rééducation du patient. Chaque séance dure environ 30 à 45 minutes et l'enfant doit recevoir des exercices à réaliser quotidiennement à la maison pendant 15 à 30 minutes selon la disponibilité des parents.

La RH correspond au type de rééducation déjà reçue par l'enfant avant l'étude; le plus souvent, il combine variablement des étirements et renforcements musculaires, une réduction du tonus musculaire (par exemple, traitement neurodéveloppemental selon le concept de Bobath), et un entraînement des habiletés motrices des membres supérieurs et inférieurs facilité par le thérapeute. Ces thérapies, impliquant des groupes limités de muscles dans des étirements ou des actions élémentaires, ciblent habituellement les muscles des membres inférieurs, parfois ceux des membres supérieurs et plus rarement ceux du tronc. Pendant la période de RH de l’étude pour le groupe PC1, le ou la kinésithérapeute propose aux parents, selon leur disponibilité et leur capacité, de réaliser quotidiennement à l’enfant, pendant 15 à 30 minutes, une sélection adaptée des étirements et actions élémentaires qu’il ou elle réalise en cabinet.

Le programme RAIT n’est pas basé sur le renforcement des muscles élémentaires du tronc, mais sur l'amélioration du contrôle postural et de l'équilibre de l'ensemble du corps, y compris le tronc et les autres muscles affectés, au moyen d'actions réalisées de manière autonome par l’enfant dans des postures intermédiaires impliquant le tronc (24). Ainsi, le principe de cette approche est, lors d'actions autonomes dans des postures intermédiaires, d'exploiter le contrôle automatique fondamental du soutien postural et de l'équilibre pour améliorer l'utilisation des muscles affectés dans le soutien et l'équilibre non seulement lors de ces actions mais aussi lors de toutes les tâches posturales et locomotrices, une approche originale proposée pour la rééducation des patients victimes d'un accident vasculaire cérébral (44). L'enfant doit contrôler son équilibre au cours de diverses actions volontaires, à partir de postures intermédiaires telles qu’alterner de façon répétée la posture à quatre pattes et la posture dite « en cobra » ou osciller le corps d’avant en arrière depuis la posture dite « chien tête en bas » (voir figure 5). Ces actions autonomes sont moins difficiles que la station debout et la marche mais des effets bénéfiques sur ces dernières sont attendus. À partir de postures intermédiaires, l'enfant effectue également des mouvements plus difficiles du tronc, nécessitant la dissociation des mouvements des ceintures scapulaire et pelvienne ou une réduction de la lordose lombaire. Les objectifs de rééducation des membres supérieurs et inférieurs définis avant l'étude et suivis lors de la RH (par exemple, étirements des ischio-jambiers, étirements des fléchisseurs plantaires et extension du poignet) sont inclus dans certaines des activités de la RAIT. Par exemple, à partir d'une position à quatre pattes, les poignets en extension, l'enfant lève les genoux, ce qui permet d’obtenir des étirements des ischio-jambiers et des fléchisseurs plantaires tout en sollicitant le tronc et l’ensemble du corps pour gérer son équilibre. Enfin, chaque enfant reçoit une sélection d’activités à réaliser quotidiennement à la maison.

Figure 5 : Exemples d’activités posturales impliquant le tronc : alterner de façon répétée la posture à quatre pattes (A) et la posture dite « en cobra » (B) ; osciller le corps d’avant en arrière depuis la posture dite « chien tête en bas » (C). Les images n'ont pas été gardées en raison de l'absence d'autorisation parentale.

- 1. Description des évaluations réalisées au cours de la recherche

Une évaluation de la fonction motrice sera réalisée au CMPRE au début de l’étude et 3, 6 et 12 mois plus tard pour évaluer l’effet du traitement. Cette évaluation comporte des évaluations cliniques et une évaluation instrumentale de la marche et de la station debout.

- - 1. Evaluations cliniques

Plusieurs évaluations cliniques seront réalisées par un kinésithérapeute :

- - - 1. Bilan neuro-orthopédique

Un thérapeute réalisera un bilan neuro-orthopédique des membres inférieurs et supérieurs. Cet examen consiste à mesurer, (i) à l’aide d’un goniomètre, les amplitudes de mouvement des principales articulations dans un ou plusieurs plans, (ii) la force musculaire des grands groupes musculaires en demandant au sujet de mobiliser une articulation contre résistance et (iii) la spasticité des principaux muscles, en mobilisant une articulation à vitesse lente puis rapide pour éliciter un réflexe d’étirement. Ce bilan dure environ 45 minutes.

- - - 1. EMFG-66-SI

L’Evaluation Motrice Fonctionnelle Globale 66 (EMFG-66) est un score clinique standardisé comportant 66 items qui permet d’évaluer la fonction motrice globale et leur évolution dans le temps des enfants atteints de paralysie cérébrale (45). L’EMFG-66-SI est une méthode de cotation plus rapide (environ 20 à 30 minutes versus 60 à 80 minutes) et validée de l’EMFG-66, utilisant 15 à 39 items (41,46).

- - - 1. L’Échelle Clinique Précoce de l’Équilibre (ECPE)

C’est une échelle clinique en 13 items qui permet d’évaluer la stabilité posturale (capacité d’équilibre) d’enfants atteints de paralysie cérébrale, avec deux sous échelles : une dédiée au contrôle postural de la tête et du tronc, une dédiée au contrôle postural assis et debout. Cette échelle est validée pour des enfants de 1,5 à 11 ans, quel soit le niveau de GMFCS (40,47). Le score optimal est de 100. La passation de cette échelle prend environs 15 minutes.

- - - 1. Questionnaire du rapport familial du Système de Classification de la Fonction Motrice Globale (GMFCS)

Ce questionnaire destiné aux parents, basé sur les mouvements volontaires de l’enfant pour la station assise, les transferts et la mobilité, permet de situer dans une classification à 5 niveaux la sévérité de la paralysie cérébrale de l’enfant. La passation du questionnaire est d’environ 2 minutes.

- - - 1. Questionnaire « Tendre la main »

C’est un questionnaire destiné aux parents afin d’évaluer les capacités du membre supérieur et de la main dans différentes situations fonctionnelles de leur enfant ayant une PC (2023 Ma). Ce questionnaire, validé pour des enfants avec PC à partir de l’âge de 2 ans, permettra d’évaluer l’amélioration attendue des fonctions de la main et du membre supérieur liée à la RAIT. La passation du questionnaire est d’environ 15 minutes.

- - 1. Analyse instrumentale de la marche et de la station debout

La piste d’analyse de la marche Zeno (figure 2) est un tapis constitué de capteurs de pression qui permet de recueillir les paramètres spatio-temporels de la marche et le déroulé des empreintes des pieds. Ces paramètres sont communément utilisés pour analyser la locomotion, identifier des troubles de la marche et évaluer l’effet d’interventions thérapeutiques. Il est demandé au patient de marcher sur la piste en faisant des allers-retours.

Quatre centrales inertielles (figure 6) seront utilisées en complément de la piste de marche, placées sur la peau avec un adhésif double-face hypoallergénique : un sur le sternum, un en regard de la 5^ème^ vertèbre lombaire (L5) et un sur chaque pied. Cela permettra de relever, en début d’appui, les pics de décélération de la partie supérieure du tronc (sternum) et de la partie inférieure du tronc proche du centre de masse (L5) et de mesurer l’orientation transversale des pieds au cours de la marche.


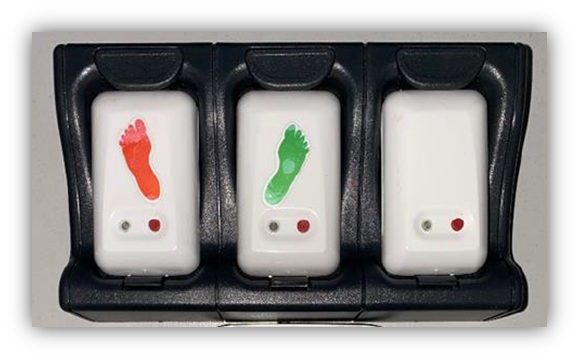


Figure 6 : exemple de centrales inertielles mTest^3^ (mHealth Technology) utilisées.

Deux caméras vidéo, une de profil et une de face, permettront d’analyser la cinématique de marche à l’aide du score visuel de marche d’Edinbourg, validé pour les enfants ayant une paralysie cérébrale (42).

L’analyse de la marche, à vitesse spontanée, réalisée en 10 minutes environ, nous permettra de recueillir les variables suivantes :

- Le pic de décélération antérieure du sternum en début d’appui (les 25 premiers % du cycle de marche), dont nous avons montré que la valeur élevée était réduite après RAIT chez l’enfant PC de 5 à 12 ans (24), constituera le critère de jugement principal.
- Le pic de décélération vers le bas de L5 en début d’appui, dont nous avons montré que la valeur élevée était réduite après RAIT chez l’enfant PC de 5 à 12 ans (24), complète la variable principale pour le comportement dynamique du tronc à la marche.
- L’index de variabilité de la marche (eGVI), récemment publié, est un score composite basé sur 9 paramètres spatio-temporels qui quantifie la distance entre la quantité de variabilité observée dans un groupe de référence asymptomatique et la quantité de variabilité observée chez le patient (38,39). Cet index évalue l’instabilité au cours de la marche et le risque de chute. Il est habituellement élevé chez l’enfant avec PC (48,49).
- La largeur de cycle (entre les 2 pieds), élevée chez l’enfant avec PC, est une des stratégies pour réduire le risque de chute en cas de marche instable (50).
- L’appui antérieur du pied lors du 1er double-appui, est une variable obtenue sur une piste de marche à capteurs de pression relative, définie par le ratio entre la pression intégrée du demi-pied antérieur et la pression intégrée de l’ensemble du pied lors du 1er double-appui. Cette variable sera d’autant plus élevée que l’appui du pied au sol se fera par l’avant pied, comme chez l’enfant PC, et d’autant plus faible que l’appui du pied se fera par le talon, comme chez l’enfant à DT. Cette variable, développée à l’occasion de cette étude, est liée au déplacement antérieur précoce du centre de pression fréquemment observé chez l’enfant avec PC (51).
- Le score visuel de marche d’Edinbourg (2003 Read), est un score développé pour les enfants ayant une paralysie cérébrale, évaluant l’importance des déviations cinématiques par rapport à des enfants au développement typique.

L’analyse de la station debout sera réalisée en demandant à l’enfant de se tenir debout et immobile pendant 30 secondes, 3 fois. L’analyse du déplacement de la centrale inertielle placée au niveau L5 grâce au logiciel mSway de mHealth, permettra d’estimer la vitesse moyenne et l’ellipse à 95 % de la surface de déplacement horizontal du centre de masse corporel (ref).

- 1. Résultats attendus

*Pour les enfants avec PC par rapport aux enfants à DT*, il est attendu :

- une valeur des pic de décélération vers l’avant du sternum et vers le bas de L5, en début d’appui, plus importante

- des valeurs de la largeur de cycle et de l’eGVI plus importantes

- un appui antérieur du pied lors du 1er double-appui plus important

- des valeurs de la vitesse moyenne et de la surface de déplacement du centre de masse plus importantes

- une valeur du score visuel de marche d’Edinbourg plus importante

- des scores de l’ECPE, de l’EMFG-66-SI et du questionnaire « tendre la main » moins importants.

*Chez les enfants avec PC*, l’effet attendu après RAIT est une amélioration des critères de jugement :

- diminution du pic de décélération vers l’avant du sternum et vers le bas de L5 en début d’appui

- diminution de la largeur de cycle, de l’eGVI et de l’appui antérieur du pied lors du 1er double-appui

- diminution du score visuel de marche d’Edinbourg

- diminution de la vitesse moyenne et de la surface de déplacement du centre de masse

- augmentation des scores l’ECPE, de l’EMFG-66-SI et du questionnaire « tendre la main ».

- 1. Plan d’analyse statistique

Les analyses statistiques seront réalisées dans le cadre de modèles linéaires généraux, l’échantillon global (N=64) et pour chaque groupe (n=32) étant a priori suffisant pour l’application de telles procédures. Les conditions d’application pour effectuer de tels tests seront préalablement vérifiées et notamment la répartition normale des résidus (à l’aide de graphes quantile-quantile et de tests de Shapiro-Wilk) et l’homogénéité de la variance (tests de Levene). Dans le cas où les données ne respecteraient pas ces conditions, des équivalents non paramétriques seront utilisés. Lors de la première phase, l’effet du groupe (Enfants avec PC vs. Enfants à DT) sera analysé à l’aide de procédures multivariées d’analyse de la variance (MANOVA) sur les variables dépendantes (VD) cinématiques (largeur de cycle), dynamiques (décélérations du sternum et de L5 et position antérieure du pied lors du 1er double-appui) et cliniques (ECAB, EMFG-66-SI et questionnaire « tendre la main ») et une analyse de régression linéaire multiple sera réalisée en choisissant, pour variable à expliquer, la position antérieure du pied lors du 1er double-appui et, pour régresseurs, les décélérations du sternum et de L5 en début d’appui. Par ailleurs, les effets de la rééducation spécifique (2 Groupes x 4 périodes) seront testés à l’aide du même type de procédures multivariées (MANOVA) et d’un plan mixte 2 Groupes [PC1 vs. PC2] × 4 Périodes [M0, M3, M6, M12], en s’intéressant tout particulièrement à l’interaction des facteurs Groupe × Période. Si cette dernière est significative, l’analyse des comparaisons locales à l’aide de procédures post-hoc (tests de Tukey) nous permettra d’examiner -1- la significativité de la rééducation (différence significative entre les deux groupes à M3 avec réduction du pic de décélération du sternum en début d’appui pour le groupe qui a suivi la RAIT entre M3 et M0, -2- la reproductibilité de l’effet de la RAIT : différences similaires entre M3 et M6 pour le groupe RH-RAIT et entre M0 et M3 pour le groupe RAIT-RAIT, -3- l’accentuation de l’effet de la RAIT lorsqu’elle poursuivie : entre M6 et M0 versus entre M3 et M0 et entre M12 et M0 versus entre M6 et M0 pour le groupe RAIT-RAIT et entre M12 et M3 versus entre M6 et M3 pour le groupe RH-RAIT.

- 1. Sorties d’étude et critère d’arrêt de l’étude

Les parents de l’enfant participant sont libres de faire sortir leur enfant de l’étude à tout moment sans nécessité de donner de raisons. Il sera également mis fin au protocole dans le cas de l’apparition au cours de l’étude d’un impératif socio-médical imposant l’arrêt de l’étude. Dans une telle situation, l’évaluation finale, si cela est possible, sera réalisée au moment de l’arrêt de l’étude.

L’étude sera arrêtée lors de la dernière visite du dernier patient en cours.

Au terme de l’étude, le promoteur en informe, dans un délai de 90 jours suivant la fin de l’étude, le CPP et les centres investigateurs.

Le promoteur peut décider de mettre fin prématurément à l’étude. Le promoteur doit alors en informer dans un délai de 15 jours le CPP, et indiquer les raisons qui motivent cet arrêt. En cas d’arrêt prématuré de l’étude, les investigateurs doivent en informer rapidement les patients participants et leur assurer un suivi approprié.

- 1. Durée de la recherche et indemnisation

Durée de la participation du patient au protocole : 12 mois

Durée de la période d’inclusion : 30 mois

Durée prévisionnelle de la recherche (inclusion + analyse) : 54 mois

Il n’y a pas d’indemnisation prévue.

- 1. Période d’exclusion et exclusivité

Période d’exclusion : pendant toute la durée de l’étude, le participant à cette étude ne pourra pas participer à une autre recherche impliquant la personne humaine.

Exclusivité : Le participant ne peut participer à aucune autre recherche de type interventionnelle pendant celle-ci.

1. Bénéfices et risques
   1. Bénéfices pour le participant

Les enfants atteints de PC participants à cette étude bénéficieront tous d’une rééducation RAIT pendant 9 ou 12 mois. Si la RAIT montre les mêmes effets que dans notre précédente étude chez des enfants âgés de 5 à 12 ans, les bénéfices directs attendus de la rééducation RAIT sont un meilleur contrôle de l’équilibre au cours des stations assise et debout et une meilleure dynamique de marche (décélérations du tronc et dynamique des pieds). De plus, en cas d’anomalies motrices d’un membre supérieur, il est aussi attendu une meilleure utilisation du membre supérieur et de la main (extension associée des coude, poignet et doigts, dextérité manuelle), ce qui est issu d’une expérience clinique non publiée.

Les kinésithérapeutes ainsi que les parents des patients pourront poursuivre cette prise en charge même après la fin de l’étude.

- 1. Risques et contraintes pour le participant

Il n’y a pas de risque majeur pour le participant concernant les traitements et les évaluations réalisés. La réalisation d’activités posturales dans lesquelles des muscles et tendons sont allongés pourraient être initialement douloureux pour ces derniers. Cependant, la douleur des muscles et tendons, si elle survenait, serait faible et transitoire car l’allongement des muscles et tendons serait progressif d’autant plus que c’est l’enfant qui réalise lui-même les activités posturales.

Les contraintes liées à l’étude sont assez importantes. En effet, les activités posturales RAIT ou, le cas échéant, une sélection adaptée d’exercices liés à la rééducation habituelle, prescrite par le kinésithérapeute, doivent être réalisées régulièrement, tous les jours, pendant au moins 15 à 30 minutes (en une ou plusieurs fois), les séances à domicile étant complémentaires de celles réalisées chez le kinésithérapeute. Les séances à domicile impliqueront la présence d’un adulte auprès de l’enfant pour l’encadrer dans ses réalisations.

Les activités posturales ou exercices de rééducation habituelle réalisés par l’enfant à domicile seront à inscrire sur un journal de suivi afin que l’évolution de ses réalisations puisse être suivie par le kinésithérapeute et par l’équipe du CMPRE. Une personne de l’équipe du CMPRE contactera les parents en téléconsultation une à deux fois entre chaque évaluation de la fonction motrice au CMPRE afin de répondre à leurs interrogations pour réaliser au mieux cette étude. Il y aura au total 4 sessions au CMPRE, au début de l’étude puis à 3, 6 et 12 mois, chacune durant plusieurs heures (2 à 4 heures) réparties sur une journée, incluant l’évaluation de la fonction motrice et une consultation médicale au cours de laquelle le point sera fait sur les activités posturales.

1. Evaluation de la sécurité
   1. Evènements indésirables
      1. Définitions

Evènement indésirable : toute manifestation nocive survenant chez une personne qui se prête à une recherche impliquant la personne humaine, que cette manifestation soit liée ou non à la recherche ou au produit sur lequel porte cette recherche.

Effet indésirable : l’effet indésirable d’une recherche correspond à tout évènement indésirable dû à la recherche.

Faits nouveaux : toute nouvelle donnée pouvant conduire à une réévaluation du rapport des bénéfices et des risques de la recherche ou du produit objet de la recherche, à des modifications dans l'utilisation de ce produit, dans la conduite de la recherche, ou des documents relatifs à la recherche, ou à suspendre ou interrompre ou modifier le protocole de la recherche ou des recherches similaires. Pour les essais portant sur la première administration ou utilisation d'un produit de santé chez des personnes qui ne présentent aucune affection : tout effet indésirable grave.

- - 1. Evènement(s) indésirable(s) grave(s)

Dans le cadre de cette étude, indépendamment de l’intensité ou de la durée du traitement, seront considérés comme grave (EIG), tout évènement ou effet indésirable qui :

- Entraîne la mort
- Met en danger la vie du participant
- Nécessite une hospitalisation ou une prolongation d’hospitalisation
- Provoque un handicap ou une incapacité importante ou durable
- Se traduit par une anomalie ou une malformation congénitale
- Se traduit par une situation médicalement significative

Dans ce protocole, ne seront pas considérés comme grave (EIG) :

- Un évènement entraînant un passage transitoire à la consultation d’un hôpital, à un service de porte ou à un hôpital de jour,
  - Les hospitalisations (plus d’une nuit sur place) ou prolongations d’hospitalisation motivées par les raisons suivantes :
  - Les hospitalisations programmées pour des interventions ou traitements de routine faisant partie d’un programme de surveillance ou de thérapie défini à l’avance,
  - Les hospitalisations pour exploration non liées à une modification de l’état du participant,
  - Une hospitalisation de confort ou pour raisons sociales (ex : hospitalisation d’une personne âgée en relation de dépendance du conjoint qui vient d’être hospitalisé).
  - Une hospitalisation élective non associée à une aggravation de l’état clinique et non liée à l’objectif de l’étude clinique et ayant lieu au cours de l’étude clinique (ex : chirurgie esthétique).

De plus, seront considérés comme inattendus, les effets indésirables graves dont la nature, la sévérité ou l’évolution ne concordent pas avec les informations relatives aux produits, actes pratiqués et méthodes utilisées au cours de la recherche.

- 1. Conduite à tenir

Tous les événements indésirables survenant au cours de l'étude qu'ils soient constatés par les médecins ou rapportés par les patients et quelle que soit l'imputabilité supposée, devront être notés dans le cahier d’observation et suivis de manière appropriée.

Dans la mesure du possible, il devra être rapporté pour chaque événement indésirable :

1. Sa description ou son diagnostic,
2. Sa durée (dates de début et de fin),
3. Sa sévérité (légère, modérée, sévère),
4. Sa relation au produit à l’essai (liée/non liée),
5. La (ou les) mesure(s) prise(s) et l’évolution.

Le degré de sévérité d’un événement indésirable est défini qualitativement par l’intensité de l’événement telle qu’elle est appréciée par l’investigateur ou signalée à ce dernier par le patient. Le degré de sévérité n’est pas le reflet de la gravité clinique de l’événement et l’évaluation de l’intensité doit être faite indépendamment de l’évaluation de l’imputabilité au produit ou de la recherche. Il représente seulement le degré ou l’importance de la gêne ou du cas (par exemple, nausée sévère, accident vasculaire cérébral léger).

Il est évalué selon les critères suivants :

**Echelle des degrés de sévérité des événements indésirables.**

| **1** | Léger | Symptôme(s) à peine visible(s) pour le patient ou n’entraînant pas d’inconfort. L’EI n’a pas d’effet comportemental ou fonctionnel. Un traitement du (des) symptôme(s) n’est généralement pas nécessaire. |
| --- | --- | --- |
| **2** | Modéré | Symptôme(s) d’une sévérité suffisante pour incommoder le patient. L’activité quotidienne est modifiée. Le traitement des symptômes peut être nécessaire. |
| **3** | Sévère | Symptôme(s) d’une sévérité suffisante pour incommoder sérieusement le patient. La sévérité peut entraîner l’arrêt du traitement. Le traitement des symptômes peut être nécessaire. |

La relation entre la réalisation de la mesure selon la méthode à l’étude et la survenue de l’événement indésirable ne peut appartenir qu’à deux catégories selon l’opinion de l’investigateur : non suspectée ou suspectée.

**Relation entre les événements indésirables et la réalisation de la mesure**

| **0** | Non liée | La relation chronologique entre la survenue de l’événement clinique par rapport à la réalisation de la mesure rend improbable tout lien de causalité, ou d’autres médicaments, actes thérapeutiques ou conditions sous-jacentes fournissent une explication suffisante à l’événement observé. |
| --- | --- | --- |
| **1** | Liée | La relation chronologique entre la survenue de l’événement clinique par rapport à la réalisation de la mesure rend possible un lien de causalité. De plus, d’autres médicaments, actes thérapeutiques ou conditions sous-jacentes ne fournissent pas d’explication suffisante à l’événement observé. |

Le suivi d'un événement indésirable sera effectué, même après la fin du protocole de recherche, si l'événement indésirable persiste après la fin de la recherche.

Ce suivi sera effectué jusqu'à ce que l'événement disparaisse ou se stabilise à un niveau jugé comme acceptable par le médecin et le moniteur de la recherche.

Pour tout événement indésirable, le médecin devra chercher à obtenir toute information permettant de savoir s'il s'agit d'un événement indésirable grave.

- - 1. Conduites à tenir en cas d’évènement indésirable grave

Les coordonnées des personnes à contacter en cas de survenue d’un évènement indésirable grave sont fournies aux thérapeutes participants à la recherche dans les cahiers de suivi et d’observation.

- - - 1. Rôle(s) de l’investigateur

Afin d'assurer la sécurité du patient, tout événement indésirable grave qu’il soit ou non suspecté, par l’investigateur, d'être en rapport avec la recherche ou le produit à l’étude, devra être déclaré à l’aide du formulaire de déclaration d’événement indésirable grave et transmis **par mail** à l’Institut Régional de Médecine Physique et de Réadaptation de Nancy ([jonathan.pierret@ugecam.assurance-maladie.fr](mailto:jonathan.pierret@ugecam.assurance-maladie.fr)), établissement de l’UGECAM du Nord-Est, **sans délai à compter du jour où il en prend connaissance**. La « Fiche de Déclaration d’un événement Indésirable Grave » présente dans le cahier d’observation doit être remplie, signée puis envoyée, éventuellement par voie dématérialisée, à l’investigateur coordonnateur : Christian Beyaert (christian.beyaert@univ-lorraine.fr), et au représentant du promoteur, Jonathan Pierret (jonathan.pierret@ugecam.assurance-maladie.fr)

Attention, l’anonymat du patient doit être préservé : le représentant du promoteur ne devra pas disposer d’informations lui permettant d’identifier le patient. Ainsi, il incombe à l’investigateur principal de s’assurer à ce que la fiche d’EIG soit traitée de manière à assurer l’anonymat du patient concerné.

L’investigateur suit les participants ayant présenté un évènement indésirable grave jusqu’à ce que l’événement soit considéré comme résolu. L’envoi de la fiche de déclaration est suivi d’un rapport détaillé contenant tous les résultats d’examens complémentaires et/ou de compte rendus d’hospitalisation, transmis dès que l’information est disponible. Toute information nouvelle est transmise au promoteur.

- - - 1. Rôle du promoteur

L’Institut Régional de Réadaptation de Nancy

- Reverra les formulaires d’événements ou effets indésirables graves transmis par l’investigateur ;
- Évaluera la relation possible avec le produit à l’étude ou la recherche et le caractère attendu ;
- Demandera si nécessaire des informations complémentaires à l’investigateur ;
- Complétera la partie réservée au promoteur sur les formulaires d’événements indésirables graves ou effets indésirables graves ;
- Conformément à l’article R1123-59 du code de la santé publique, le promoteur informera sans délai l'autorité compétente et le comité de protection des personnes des faits nouveaux définis au 12° de l'article [R. 1123-46](https://www.legifrance.gouv.fr/affichCodeArticle.do?cidTexte=LEGITEXT000006072665&idArticle=LEGIARTI000006908425&dateTexte=&categorieLien=cid) et, le cas échéant, des mesures prises.
- Le promoteur transmettra également à tous les investigateurs concernés les informations susceptibles d’affecter la sécurité des personnes se prêtant à l’essai, notamment toute information pertinente relative aux suspicions d’effet indésirable grave et inattendu et aux événements indésirables graves susceptibles d’être liés à la procédure de mise en œuvre du dispositif médical étudié qui pourraient avoir un impact défavorable sur la sécurité des personnes.

1. Gestion des données
   1. Droit d’accès aux données

L’investigateur s’engage à respecter le protocole et à l’appliquer en tenant compte des bonnes pratiques cliniques.

Il s’engage à laisser libre l’accès aux données de l’étude au promoteur, moniteurs, auditeurs ou personnes représentant les Autorités de Santé.

A la fin de l’étude, si le volontaire le souhaite, il pourra être informé des résultats globaux de la recherche (Article L1122-1, dernier alinéa). Le participant devra faire la demande par écrit au coordonnateur.

- 1. Données sources

Les documents sources sont définis comme tout document ou objet original permettant de prouver l’existence ou l’exactitude d’une donnée ou d’un fait enregistré au cours de l’essai clinique. Elles comprennent les documents originaux, les données et les dossiers à partir desquels les données de l’étude concernant le sujet sont reportées dans le cahier d’observation. Celles-ci incluent, entre autres, le dossier médical, les rapports et résultats d’examen, les questionnaires, la correspondance médicale. Les résultats des investigations ou estimations inclus dans le cahier d’observation sont considérés comme des données sources.

Les données seront retranscrites lisiblement dans le cahier d’observation par les investigateurs ou leur(s) collaborateur(s). Une trace de toutes les modifications apportées aux cahiers d’observations sera conservée. Ces traces doivent permettre de connaître, pour toute modification, la valeur antérieure et la personne ayant procédé à la modification.

L’absence d’une donnée est expliquée (donnée manquante, mesure non réalisée, non applicable, ne sait pas).

Ces cahiers sont remplis sous la responsabilité de l’investigateur du centre et des co-investigateurs qui doivent veiller à l’exactitude des données recueillies. L’identité de la personne, ayant rempli le cahier d’observation, sera indiquée. Pour attester son accord avec les données figurant dans le cahier d’observation, l’investigateur paraphera chaque page du cahier d’observation.

- 1. Anonymats des participants

L’anonymat des participants sera assuré en lui associant une référence alphanumérique unique : par exemple, pour le premier participant, cette référence sera la suivante : FLA-001 Cette référence sera utilisée pour nommer les fichiers comportant les données à analyser et effectuer les calculs et les études statistiques. La liste correspondante entre les références et les participants sera reportée dans le dossier de l’étude.

Des enregistrements vidéo et photo pourront être pris, sous réserve de l’acceptation du participant. L’anonymat du participant sera assuré en masquant son visage ainsi que tout signe distinctif. Nous utiliserons pour cela le logiciel de montage vidéo KineMaster qui permet d’appliquer une mosaïque sur l’image. Concernant les photos, l’anonymat sera assuré en floutant le visage du participant, ainsi que tout signe distinctif, via le logiciel Paint.net.

- 1. Confidentialité

Conformément aux dispositions de l’article R. 5120 du Code de la Santé Publique, l’investigateur et toute personne appelée à collaborer aux essais sont tenus au secret professionnel, en particulier en ce qui concerne notamment la nature des produits étudiés, les essais, les personnes qui s’y prêtent et les résultats obtenus sous réserve des dispositions de l’article L. 209-12 du Code de la Santé Publique.

Aussi, les essais ne feront l’objet d’aucun commentaire, oral ou écrit, sans une autorisation conjointe de l’investigateur coordonnateur et du promoteur.

- 1. Archivage

Au terme de l’étude, tous les documents en relation avec l’étude (y compris la copie des cahiers d’observation) seront archivés sur les sites d’étude ou dans une archive centralisée. Une attention particulière doit être portée à la liste permettant d’identifier les participants inclus dans l’essai et aux formulaires de consentement. Cette liste et les formulaires de consentement sont les documents les plus importants des dossiers devant être archivés par l’investigateur.

Tous les documents en relation avec l’étude devront être conservés pendant 15 ans après la fin de l’étude. A la fin de cette période, le promoteur informera les investigateurs de la fin de l’archivage.

- 1. Règles relatives à la publication au rapport final

Cette étude donnera lieu à des publications, à la rédaction de mémoires de masters et/ou de thèses.

Un résumé de ce rapport final sera transmis au CPP dans les 12 mois suivant la fin de l’étude.

1. Assurance Qualité
   1. Engagement de l’investigateur - Bonnes pratiques cliniques

L’investigateur s’engage à respecter le protocole et à l’appliquer en tenant compte des bonnes pratiques cliniques. Tout écart au protocole doit être documenté, indiquant la date et la raison, les mesures prises et les conséquences pour le sujet et l’étude. Les documents afférents sont conservés dans le dossier de l’investigateur.

L’investigateur s’engage à laisser libre l’accès aux données de l’étude au promoteur, moniteurs, auditeurs ou personnes représentant les Autorités de Santé.

- 1. Contrôle de la qualité des données

D’autre part, une fois les données saisies, des contrôles des données (contrôles de cohérence et de données manquantes) seront fait régulièrement pour les données cliniques. La fréquence de ces contrôles dépendra du rythme des inclusions et des documents récupérés par les centres investigateurs. Les données manquantes ou incohérentes feront l’objet d’un retour aux investigateurs pour complément d’information et vérification.

- 1. Audit et Inspection

Les investigateurs et le promoteur acceptent de se conformer aux exigences de ces évaluations, dans le cas où elles seraient réalisées.

L’investigateur s’engage à laisser libre l’accès aux données de l’étude au promoteur, moniteurs, auditeurs ou personnes représentant les Autorités de Santé.

1. Organisation de l’étude
   1. Comité scientifique

Le comité scientifique est piloté par le docteur Beyaert.

Les objectifs de ce comité sont définis ci-dessous. Il est chargé de la définition de l’objectif de l’étude, de la rédaction du protocole et du cahier d’observation de l’étude, et du choix des centres. Il aura également pour rôle de veiller au bon déroulement de l’étude sur le plan scientifique et logistique, de résoudre les difficultés rencontrées, de prendre toutes les décisions concernant l’étude (amendement, décision de son arrêt prématuré ou de sa prolongation, la réévaluation de l’effectif de l’étude …). Enfin il assure la surveillance dans l’analyse des résultats de l’étude et définit la stratégie de valorisation des données de l’étude à travers la validation de l’analyse statistique et la coordination de la rédaction des documents issus des résultats de l’étude. Le comité vérifiera que les conditions pour être auteur sont réunies à la fin de l’étude lors de la rédaction du manuscrit, selon les règles du protocole de recherche et/ou des recommandations du groupe de Vancouver. En cas de conflit entre investigateurs, celui-ci est soumis à l’appréciation du comité en premier lieu puis du promoteur en deuxième lieu.

1. Considérations éthiques et règlementaires
   1. Autorisation de lieu de recherche

Une Autorisation de lieu est nécessaire pour les recherches réalisées en dehors des lieux de soins ou dans des services hospitaliers lorsque ces recherches nécessitent des actes autres que ceux qu’ils pratiquent usuellement dans le cadre de leur activité. L’IRR, est un lieu de soins pour lequel les actes pratiqués dans le cadre de cette recherche seront ceux usuellement pratiqués dans le cadre de son activité.

- 1. Conduite éthique de l’étude

La planification et la conduite de cette étude sont régies par les lois françaises et européennes (notamment le Décret n° 2016-1537 (loi Jardé) du 16 novembre 2016). Cet essai ne pourra commencer que lorsque l’ensemble des dispositions légales relatives aux obligations préalables à la mise en œuvre d’une recherche impliquant la personne humaine auront été respectées. L’étude sera conduite en accord avec les principes éthiques de la déclaration d’Helsinki et les recommandations des Bonnes Pratiques Cliniques – ICH du 17 janvier 1997.

Le responsable de la recherche du promoteur pourra décider de l’arrêt complet de l’étude pour raisons médicales. De plus, le promoteur se réserve le droit de mettre fin à l’étude à tout moment si celle-ci ne peut pas être menée en accord avec le protocole.

En cas d’arrêt prématuré ou en cas de suspension de l’étude, le promoteur informera rapidement les investigateurs, ainsi que les autorités réglementaires et le C.P.P. de l’arrêt ou de la suspension de l’étude, ainsi que de la raison de cette décision.

- 1. Comité de Protection des Personnes

L’essai ne peut débuter sans avoir reçu l’autorisation du C.P.P. Le protocole débutera lors de la réception de l’avis favorable du CPP. L’autorisation devient caduque si dans un délai d’un an suivant l’autorisation, la recherche n’a pas débuté (à savoir aucune personne incluse dans le protocole).

Ni l’investigateur, ni le promoteur ne peuvent modifier ce protocole sans accord préalable écrit de l’autre partie. Si des modifications substantielles doivent être apportées, celles-ci doivent faire l’objet d’un amendement au protocole.

Cet amendement sera appliqué lorsqu’il aura reçu la double autorisation du C.P.P.

- 1. Responsabilités de l’investigateur

L'investigateur s'engage à ce que cette étude soit réalisée en conformité avec la loi n° 2004-806 du 9 août 2004 relative à la politique de santé publique et à ses décrets d’application, la déclaration d'Helsinki, les Bonnes Pratiques Cliniques. Toutes les données, tous les documents et rapports pourront faire l'objet d'audits et d'inspections réglementaires sans que puissent être opposé le secret médical.

Pour rappel, l’investigateur informera les volontaires des objectifs et des contraintes de l'étude, de leurs droits de refuser de participer à l'étude ou de la quitter à tout moment. Lorsque l'information aura été donnée au sujet, l'investigateur se sera assuré qu'il a bien compris les implications de la participation à l'étude, son consentement écrit sera recueilli par un des investigateurs en deux exemplaires originaux. Comme spécifié précédemment, un exemplaire original du formulaire d'information et du consentement signé sera remis au sujet, l'autre exemplaire du consentement sera conservé par l’investigateur.

Toutes les informations recueillies sont confidentielles et ne pourront être divulguées. L'investigateur s'assurera que l'anonymat de chaque volontaire participant à l'étude est garanti. Aucune information permettant l'identification des personnes ne sera communiquée à des tiers autres que ceux, représentant du promoteur et du Ministère de la Santé, réglementairement habilités à détenir cette information (et qui sont tenus au secret professionnel).

- 1. Responsabilités du promoteur

Conformément à la loi n° 2004-806 du 9 août 2004 relative à la politique de santé publique et à ses décrets d’application (notamment au décret n° 2016-1537 du 16 novembre 2016 relatif aux recherches impliquant la personne humaine), le promoteur s’engage à réaliser l’ensemble des opérations qui lui incombent :

**Assurance :** le promoteur de l’étude, souscrit pour toute la durée de l'étude une assurance garantissant sa propre responsabilité civile ainsi que celle de tout intervenant impliqué dans la réalisation de l'essai, indépendamment de la nature des liens existant entre les intervenants et le promoteur.

**Comité de Protection des Personnes :** le promoteur sollicitera l’avis d’un Comité de Protection des Personnes. La recherche ne pourra être mise en œuvre qu’après l’avis favorable du CPP.

**Commission Nationale Informatique et Liberté :** Les informations recueillies lors de cette étude pourront faire l'objet d'un traitement informatique.

Cette étude entre dans le cadre la méthodologie MR-001. Conformément à la délibération n° 2018-153 du 3 mai 2018 portant homologation d'une méthodologie de référence relative aux traitements de données à caractère personnel mis en œuvre dans le cadre des recherches dans le domaine de la santé avec recueil du consentement de la personne concernée (MR-001) le Promoteur a adressé à la CNIL un engagement de conformité à la méthodologie MR-001 à la date du 25/01/2021 sous le numéro 2220869.

**Modification substantielle :** Après le commencement de l’étude, toute modification substantielle du protocole à l'initiative de l’investigateur sera soumise au promoteur ; celui-ci doit obtenir, préalablement à sa mise en œuvre, un avis favorable du CPP

- 1. Déclaration des fichiers

Le recueil et le traitement des données seront réalisés conformément à la méthodologie de référence MR-001 modifiée de la CNIL. La base juridique retenue est l’intérêt public. Les données médicales concernant les participants font l'objet d'un traitement informatique et ne seront transmises qu'au promoteur, ainsi que le cas échéant aux autorités sanitaires habilitées, dans des conditions garantissant leur confidentialité. Les participants pourront exercer leurs droits d'accès, de rectification, d’opposition et de suppression auprès de l’investigateur en charge de la recherche, ou par l’intermédiaire d’un autre médecin désigné à cet effet.

1. Bibliographie

1. Graham HK, Rosenbaum P, Paneth N, Dan B, Lin JP, Damiano DL, et al. Cerebral palsy. Nat Rev Dis Primers. 07 2016;2:15082.

2. Pierret J, Beyaert C, Paysant J, Caudron S. How do children aged 6 to 11 stabilize themselves on an unstable sitting device? The progressive development of axial segment control. Human Movement Science. juin 2020;71:102624.

3. Pin TW, Butler PB, Cheung HM, Shum SLF. Relationship between segmental trunk control and gross motor development in typically developing infants aged from 4 to 12 months: a pilot study. BMC Pediatr. déc 2019;19(1):425.

4. Pin TW, Butler PB, Cheung HM, Shum SLF. Longitudinal Development of Segmental Trunk Control in Full Term and Preterm Infants- a Pilot Study: Part II. Developmental Neurorehabilitation. 2 avr 2020;23(3):193‑200.

5. Saavedra SL, Woollacott MH. Segmental Contributions to Trunk Control in Children With Moderate-to-Severe Cerebral Palsy. Archives of Physical Medicine and Rehabilitation. juin 2015;96(6):1088‑97.

6. Pierret J, Caudron S, Paysant J, Beyaert C. Impaired postural control of axial segments in children with cerebral palsy. Gait & Posture [Internet]. 8 mars 2021 [cité 9 mars 2021]; Disponible sur: https://www.sciencedirect.com/science/article/pii/S0966636221000977

7. Heyrman L, Feys H, Molenaers G, Jaspers E, Monari D, Nieuwenhuys A, et al. Altered trunk movements during gait in children with spastic diplegia: Compensatory or underlying trunk control deficit? Research in Developmental Disabilities. 1 sept 2014;35(9):2044‑52.

8. Attias M, Bonnefoy-Mazure A, Lempereur M, Lascombes P, De Coulon G, Armand S. Trunk movements during gait in cerebral palsy. Clinical Biomechanics. janv 2015;30(1):28‑32.

9. Wallard L, Dietrich G, Kerlirzin Y, Bredin J. Balance control in gait children with cerebral palsy. Gait & Posture. mai 2014;40(1):43‑7.

10. Kim CJ, Son SM. Comparison of Spatiotemporal Gait Parameters between Children with Normal Development and Children with Diplegic Cerebral Palsy. J Phys Ther Sci. 2014;26(9):1317‑9.

11. Hsue BJ, Miller F, Su FC. The dynamic balance of the children with cerebral palsy and typical developing during gait. Part II: Instantaneous velocity and acceleration of COM and COP and their relationship. Gait & Posture. avr 2009;29(3):471‑6.

12. Saether R, Helbostad JL, Adde L, Brændvik S, Lydersen S, Vik T. Gait characteristics in children and adolescents with cerebral palsy assessed with a trunk-worn accelerometer. Research in Developmental Disabilities. juill 2014;35(7):1773‑81.

13. Meyns P, Kerkum YL, Brehm MA, Becher JG, Buizer AI, Harlaar J. Ankle foot orthoses in cerebral palsy: effects of ankle stiffness on trunk kinematics, gait stability and energy cost of walking. European Journal of Paediatric Neurology. 2020;

14. Armand S, Watelain E, Mercier M, Lensel G, Lepoutre FX. Identification and classification of toe-walkers based on ankle kinematics, using a data-mining method. Gait & posture. 2006;23(2):240‑8.

15. Beyaert C, Pierret J, Vasa R, Paysant J, Caudron S. Toe walking in children with cerebral palsy: a possible functional role for the plantar flexors. Journal of Neurophysiology. oct 2020;124(4):1257‑69.

16. Worthen-Chaudhari L, Bing J, Schmiedeler JP, Basso DM. A new look at an old problem: Defining weight acceptance in human walking. Gait & Posture. janv 2014;39(1):588‑92.

17. Colborne GR, Wright FV, Naumann S. Feedback of triceps surae EMG in gait of children with cerebral palsy: a controlled study. Archives of physical medicine and rehabilitation. 1994;75(1):40‑5.

18. Nielsen JB, Christensen MS, Farmer SF, Lorentzen J. Spastic movement disorder: should we forget hyperexcitable stretch reflexes and start talking about inappropriate prediction of sensory consequences of movement? Exp Brain Res [Internet]. 7 mai 2020 [cité 29 mai 2020]; Disponible sur: http://link.springer.com/10.1007/s00221-020-05792-0

19. Willerslev-Olsen M, Andersen JB, Sinkjaer T, Nielsen JB. Sensory feedback to ankle plantar flexors is not exaggerated during gait in spastic hemiplegic children with cerebral palsy. J Neurophysiol. févr 2014;111(4):746‑54.

20. Lorentzen J, Willerslev-Olsen M, Hüche Larsen H, Farmer SF, Nielsen JB. Maturation of feedforward toe walking motor program is impaired in children with cerebral palsy. Brain. 1 mars 2019;142(3):526‑41.

21. Perry J, Burnfield JM. Gait analysis: normal and pathological function. 2nd. Thorofare, NJ: Slack Incorporated. 2010;

22. Correa TA, Schache AG, Graham HK, Baker R, Thomason P, Pandy MG. Potential of lower-limb muscles to accelerate the body during cerebral palsy gait. Gait & posture. 2012;36(2):194‑200.

23. Kurz MJ, Stuberg WA, DeJong SL. Mechanical work performed by the legs of children with spastic diplegic cerebral palsy. Gait & Posture. mars 2010;31(3):347‑50.

24. Pierret J, Beyaert C, Vasa R, Rumilly E, Paysant J, Caudron S. Rehabilitation of Postural Control and Gait in Children with Cerebral Palsy: the Beneficial Effects of Trunk-Focused Postural Activities. Developmental Neurorehabilitation. 23 mars 2023;1‑13.

25. Neptune RR, Kautz SA, Zajac FE. Contributions of the individual ankle plantar flexors to support, forward progression and swing initiation during walking. Journal of Biomechanics. nov 2001;34(11):1387‑98.

26. Heyrman L, Molenaers G, Desloovere K, Verheyden G, De Cat J, Monbaliu E, et al. A clinical tool to measure trunk control in children with cerebral palsy: The Trunk Control Measurement Scale. Research in Developmental Disabilities. nov 2011;32(6):2624‑35.

27. Arı G, Günel MK. A Randomised Controlled Study to Investigate Effects of Bobath Based Trunk Control Training on Motor Function of Children with Spastic Bilateral Cerebral Palsy. International Journal of Clinical Medicine. 2017;08(04):205.

28. Butler PB. A preliminary report on the effectiveness of trunk targeting in achieving independent sitting balance in children with cerebral palsy. Clinical Rehabilitation. 1998;12(4):281‑93.

29. El Shemy SA. Trunk endurance and gait changes after core stability training in children with hemiplegic cerebral palsy: A randomized controlled trial. BMR. 28 nov 2018;31(6):1159‑67.

30. ElBasatiny H, Abdelaziem A. Effect of Trunk Exercises on Trunk control, Balance and Mobility Function in Children with Hemiparetic Cerebral Palsy. IJTRR. 2015;4(5):236.

31. Numanoğlu Akbaş A, Kerem Günel M. Effects of Trunk Training on Trunk, Upper and Lower Limb Motor Functions in Children with Spastic Cerebral Palsy: A Stratified Randomized Controlled Trial. Konuralp Tıp Dergisi. 28 juin 2019;253‑9.

32. van Tittelboom V, Heyrman L, De Cat J, Algoet P, Peeters N, Alemdaroğlu-Gürbüz I, et al. Intensive Therapy of the Lower Limbs and the Trunk in Children with Bilateral Spastic Cerebral Palsy: Comparing a Qualitative Functional and a Functional Approach. JCM. 15 juin 2023;12(12):4078.

33. Martín-Valero R, Vega-Ballón J, Perez-Cabezas V. Benefits of hippotherapy in children with cerebral palsy: A narrative review. European Journal of Paediatric Neurology. nov 2018;22(6):1150‑60.

34. Moraes AG, Copetti F, Angelo VR, Chiavoloni LL, David AC. The effects of hippotherapy on postural balance and functional ability in children with cerebral palsy. Journal of Physical Therapy Science. 2016;28(8):2220‑6.

35. Mutoh T, Mutoh T, Tsubone H, Takada M, Doumura M, Ihara M, et al. Impact of Long-Term Hippotherapy on the Walking Ability of Children With Cerebral Palsy and Quality of Life of Their Caregivers. Front Neurol. 13 août 2019;10:834.

36. Santos de Assis G, Schlichting T, Rodrigues Mateus B, Gomes Lemos A, dos Santos AN. Physical therapy with hippotherapy compared to physical therapy alone in children with cerebral palsy: systematic review and meta‐analysis. Develop Med Child Neuro. févr 2022;64(2):156‑61.

37. Palisano R, Rosenbaum P, Walter S, Russell D, Wood E, Galuppi B. Development and reliability of a system to classify gross motor function in children with cerebral palsy. Developmental Medicine & Child Neurology. 29 sept 2008;39(4):214‑23.

38. Gouelle A, Mégrot F, Presedo A, Husson I, Yelnik A, Penneçot GF. The Gait Variability Index: A new way to quantify fluctuation magnitude of spatiotemporal parameters during gait. Gait & Posture. juill 2013;38(3):461‑5.

39. Gouelle A, Rennie L, Clark DJ, Mégrot F, Balasubramanian CK. Addressing limitations of the Gait Variability Index to enhance its applicability: The enhanced GVI (EGVI). Tan MP, éditeur. PLoS ONE. 1 juin 2018;13(6):e0198267.

40. McCoy SW, Bartlett DJ, Yocum A, Jeffries L, Fiss AL, Chiarello L, et al. Development and validity of the early clinical assessment of balance for young children with cerebral palsy. Developmental Neurorehabilitation. déc 2014;17(6):375‑83.

41. Avery LM, Russell DJ, Rosenbaum PL. Criterion validity of the GMFM-66 item set and the GMFM-66 basal and ceiling approaches for estimating GMFM-66 scores. Dev Med Child Neurol. juin 2013;55(6):534‑8.

42. Read HS, Hazlewood ME, Hillman SJ, Prescott RJ, Robb JE. Edinburgh Visual Gait Score for Use in Cerebral Palsy: Journal of Pediatric Orthopaedics. mai 2003;23(3):296‑301.

43. Yam WKL, Leung MSM. Interrater Reliability of Modified Ashworth Scale and Modified Tardieu Scale in Children With Spastic Cerebral Palsy. J Child Neurol. déc 2006;21(12):1031‑5.

44. Beyaert C, Vasa R, Frykberg GE. Gait post-stroke: Pathophysiology and rehabilitation strategies. Neurophysiologie Clinique/Clinical Neurophysiology. nov 2015;45(4‑5):335‑55.

45. Russell DJ, Avery LM, Rosenbaum PL, Raina PS, Walter SD, Palisano RJ. Improved Scaling of the Gross Motor Function Measure for Children With Cerebral Palsy: Evidence of Reliability and Validity. Physical Therapy. 1 sept 2000;80(9):873‑85.

46. Russell DJ, Avery LM, Walter SD, Hanna SE, Bartlett DJ, Rosenbaum PL, et al. Development and validation of item sets to improve efficiency of administration of the 66-item Gross Motor Function Measure in children with cerebral palsy: Validation of GMFM-66 Item Sets. Developmental Medicine & Child Neurology. févr 2010;52(2):e48‑54.

47. LaForme Fiss A, McCoy SW, Bartlett D, Avery L, Hanna SE, On Track Study Team. Developmental Trajectories for the Early Clinical Assessment of Balance by Gross Motor Function Classification System Level for Children With Cerebral Palsy. Physical Therapy. 1 févr 2019;99(2):217‑28.

48. Joanna M, Magdalena S, Katarzyna BM, Daniel S, Ewa LD. The Utility of Gait Deviation Index (GDI) and Gait Variability Index (GVI) in Detecting Gait Changes in Spastic Hemiplegic Cerebral Palsy Children Using Ankle–Foot Orthoses (AFO). Children. 25 sept 2020;7(10):149.

49. Prosser LA, Atkinson HL, Alfano JM, Leff M, Kessler SK, Gouelle A, et al. Normalizing step-to-step variability to age in children and adolescents with hemiplegia. Gait & Posture. oct 2022;98:6‑8.

50. Kurz MJ, Arpin DJ, Corr B. Differences in the dynamic gait stability of children with cerebral palsy and typically developing children. Gait & Posture. juill 2012;36(3):600‑4.

51. Mancinelli C, Patel S, Deming LC, Schmid M, Patritti BL, Chu JJ, et al. Assessing the feasibility of classifying toe-walking severity in children with cerebral palsy using a sensorized shoe. In: 2009 Annual International Conference of the IEEE Engineering in Medicine and Biology Society [Internet]. Minneapolis, MN: IEEE; 2009 [cité 13 sept 2023]. p. 5163‑6. Disponible sur: http://ieeexplore.ieee.org/document/5332733/

1. Annexes
   1. Annexe 1 : liste des investigateurs et des collaborateurs scientifiques

| **Investigateurs** | | | |
| --- | --- | --- | --- |
| **Prénom, nom** | **Titre/fonction** | **Numéro RPPS** | **Adresse** |
| Christian, Beyaert | PU-PH, pédiatre, investigateur coordinateur | 10002353976 | Centre de Médecine Physique et de Réadaptation pour Enfants, IRR de Nancy, 46 rue du Doyen Parisot, 54630 Flavigny-sur-Moselle |
| Fanny Dalmont | Médecin MPR, médecin investigatrice | 10101432648 | Centre de Médecine Physique et de Réadaptation pour Enfants, IRR de Nancy, 46 rue du Doyen Parisot, 54630 Flavigny-sur-Moselle |
| Marion, Birck | Médecin MPR, investigatrice | 10101658424 | Centre de Médecine Physique et de Réadaptation pour Enfants, IRR de Nancy, 46 rue du Doyen Parisot, 54630 Flavigny-sur-Moselle |

| **Collaborateurs scientifiques** | | |
| --- | --- | --- |
| **Prénom, nom** | **Titre/fonction** | **Adresse** |
| Jonathan, Pierret | PhD, Responsable Cellule de Recherche IRR | Institut Régional de Médecine Physique et de Réadaptation de Nancy, 75 Boulevard Lobau, 54042, NANCY CEDEX |
| Sandrine, Regef | Médecin MPR  Médecin coordinateur du CMPRE | Centre de Médecine Physique et de Réadaptation pour Enfants, IRR de Nancy, 46 rue du Doyen Parisot, 54630 Flavigny-sur-Moselle |
| Christelle Requena | Assistante de Recherche Clinique – UGECAM du Nord-Est | Institut Régional de Médecine Physique et de Réadaptation de Nancy, 75 Boulevard Lobau, 54042, NANCY CEDEX |
| Stella Zografou | Kinésithérapeute | Institut Régional de Médecine Physique et de Réadaptation de Nancy, 75 Boulevard Lobau, 54042, NANCY CEDEX |

- 1. Annexe 2 : liste des sites d’investigation clinique
- CMPRE de Flavigny-Sur-Moselle, Institut Régional de Médecine Physique et de Réadaptation (IRR), établissement de l’UGECAM du Nord-Est, 46 Rue du Doyen Jacques Parisot BP2, 54630 Flavigny-sur-Moselle
